# Explainable transformer framework for fast cotton leaf diagnostics and fabric defect detection

**DOI:** 10.1016/j.isci.2025.114411

**Published:** 2025-12-11

**Authors:** S M Masfequier Rahman Swapno, Anamul Sakib, Al Shahriar Uddin Khondakar Pranta, Amira Hossain, Jesika Debnath, Abdullah Al Noman, Abdullah Al Sakib, Md. Redwan Ahmed, Rezaul Haque, Abhishek Appaji

**Affiliations:** 1 Department Of Computer Science and Engineering, Bangladesh University of Business and Technology, Dhaka 1216, Bangladesh; 2 Department of Business Administration, International American University, 3440 Wilshire Blvd STE 1000, Los Angeles, CA 90010, USA; 3 Department of Computer Science, Wright State University, 3640 Colonel Glenn Hwy, Dayton, OH 45435, USA; 4 Department of Computer Science, Westcliff University, Irvine, CA 92614, USA; 5 Department of Information Technology, Westcliff University, Irvine, CA 92614, USA; 6 Department of Computer Science and Engineering, East West University, Dhaka 1212, Bangladesh; 7 Department of Medical Electronics Engineering, B.M.S. College of Engineering, Bangalore, Karnataka 560019, India; 8 Maastricht University, University Eye Clinic Maastricht, Minderbroedersberg 4-6, Maastricht, the Netherlands

**Keywords:** Plant biotechnology, Plant bioinformatics, Interaction of plants with organisms, Agricultural plant products

## Abstract

This study introduces a hybrid deep learning model that combines CNN-based hierarchical feature extraction with light-efficient vision transformer self-attention to classify multiple types of cotton leaf diseases and fabric defects. Using Explainable AI (XAI) techniques, the framework enhances interpretability, allowing domain experts to better understand the model’s decisions. Evaluated on four benchmark datasets, the proposed XCottL-FebViT achieved consistent improvements in accuracy, MCC, and F1 Score compared with leading transformer-based models, while maintaining computational efficiency through hyperparameter optimization. For CottonLeafNet and SAR-CLD, it attained training accuracies of 99.97% and 99.95%, with validation accuracies of 99.93% and 99.91%, respectively. In fabric defect classification, the model achieved 99.97% training accuracy on CottonFabricImageBD and FabricSpotDefect, with validation accuracies of 99.93% and 99.95%, respectively. A lightweight web-based application enables practical deployment for remote disease and defect detection. This work highlights the integration of interpretability, efficiency, and high performance in AI-driven agricultural and textile quality assessment.

## Highlights

• Explainable ViT framework identifies cotton leaf diseases and fabric defects
• Cross-domain model achieves high accuracy with lightweight computation
• Attention maps enhance interpretability and expert trust in predictions
• Web-based deployment enables real-time, transparent defect detection

## Graphical abstract

**Figure:**
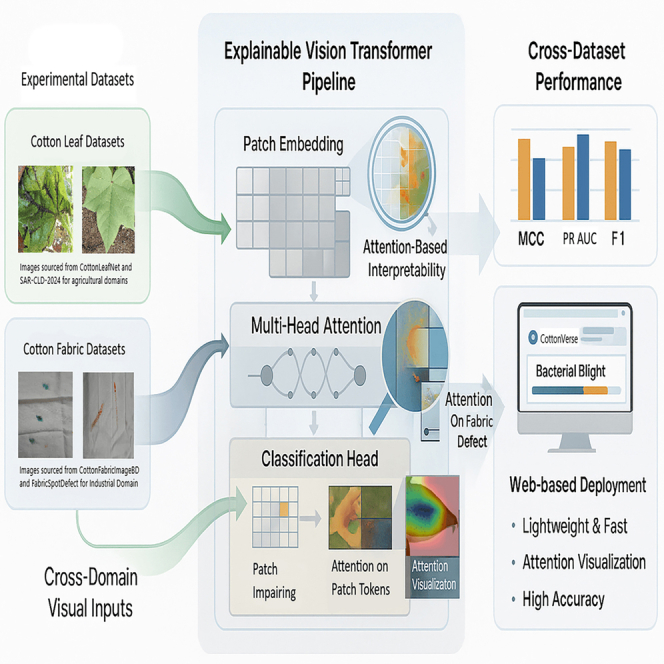

## Introduction

The textile industry primarily relies on cotton as its main cash crop, producing around 25 million metric tons annually.^1^^,^^2^ This crop supports the agricultural and manufacturing sectors, affecting the livelihoods of over 350 million people worldwide.^3^^,^^4^ However, cotton leaf diseases significantly impact crop yields each year, leading to a reduction in production by 20%–30% globally.^5^ The three most common diseases affecting cotton plants are bacterial blight, powdery mildew, and cotton boll rot, all of which harm plant health and fiber quality. Consequently, textile manufacturers incur approximately $2 billion in losses each year due to defective products.^6^ Additionally, the costs of manufacturing rise because of these defects, which occur in fabrics at rates between 5% and 8%.^7^ The immediate deployment of DL-based automated classification systems is essential to address these challenges. These systems can enhance agricultural sustainability while improving the efficiency of textile production.

Recent biological studies^8^^,^^9^ have highlighted the importance of enhancing Verticillium wilt resistance in cotton plants by targeting ROS and the SA pathway. While these findings deepen our understanding of disease resistance at the molecular level, timely detection of disease symptoms is crucial for effective management. Automated detection systems, like the one proposed in this study, can facilitate early monitoring and intervention, helping to minimize yield loss in cotton farming. In the textile industry, previous advancements have established standardized data collection and intelligent quality prediction systems for the weaving department, showcasing the potential of automation in inspection. This study builds on that foundation by combining lightweight ViT-based architectures with CNN feature extraction and explainable AI to develop a real-time, edge-deployable fabric defect classification system. The goal is to enhance quality control in weaving operations by achieving high classification accuracy and interpretability, making it practical for industrial use.

Human experts^10^ rely on visual methods for cotton farming and textile quality control during their traditional manual inspection processes. These time-consuming assessment procedures involve notable human labor, often leading to inconsistent results. Research indicates that the accuracy of disease classification through manual inspections ranges between 65% and 80%, which depends on the inspector’s experience level and external environmental conditions at the time of assessment. Additionally, the error rate for human fabric inspectors is approximately 30%, resulting in production issues and quality problems in textile manufacturing, ultimately increasing operational expenses.^11^ With the continuous rise in global manufacturing demands, traditional large-scale techniques have become unsustainable, highlighting the urgent need for AI-automated systems.

Recent studies in deep learning and computer vision have led to effective systems for detecting plant diseases and identifying fabric defects. Research by Kukadiya et al.,^12^ Peyal et al.,^13^ and Rai et al.^14^ reported accuracies over 96% using customized CNNs and optimized AlexNet variants. However, these models are limited by their dependence on small datasets and high computational costs, which hinder scalability. Ensemble learning has enhanced CNN performance, with Memon et al.^15^ achieving 98.5% accuracy through a meta-learning ensemble. Taher et al.^16^ found that deeper architectures such as VGG and ResNet, did not improve results on smaller datasets. While CNN-based models show strong pattern recognition capabilities, they often face issues such as overfitting, high computation requirements, and a lack of interpretability, making them less suitable for real-time applications.

The rise of ViT architectures has greatly enhanced agricultural disease detection accuracy. Models such as RVT by Vallabhajosyula et al.^17^ and GreenViT by Parez et al.^18^ have reached near-perfect accuracy (up to 100%) on various plant datasets, with reduced parameter counts. However, these models still require extensive training data and high-performance hardware, limiting their practical use. Qiu et al.^19^ and Baek et al.^20^ added multi-attention and patch-embedding improvements to enhance robustness in variable lighting and occlusion, but the computational demands remain high. Most ViT architectures have yet to be applied to cotton-specific tasks and struggle to balance efficiency and interpretability.

XAI frameworks prioritize transparency in model decision-making. Kamal et al.^21^ proposed DVTXAI, an explainable transformer that uses SHAP interpretability, achieving over 99% accuracy on the PlantVillage dataset but with a high inference cost. Askr et al.^22^ combined feature selection using Gray Wolf Optimization and Copula Entropy with ResNet50, providing effective interpretability while sacrificing computational efficiency. Shao et al.^23^ introduced CANnet, which enhances feature precision with receptive-field and coordinate attention mechanisms, achieving high accuracy on multi-source datasets but requiring powerful GPUs. These studies highlight that while explainability fosters user trust, current XAI frameworks are resource-intensive and not suited for low-power or potential applications.

To overcome the limitations of computationally intensive models, recent research has integrated deep learning with edge computing and IoT systems. Gao et al.^24^ employed transformer architectures and knowledge graphs, achieving a high inference speed of 49.7 FPS but struggling with generalization in varying environmental conditions. Remya et al.^25^ combined ViTs with acoustic sensing for whitefly detection, reaching 99% accuracy but facing challenges due to environmental noise. These studies highlight that while edge-oriented systems support real-time disease monitoring, they need to improve robustness against data variability, sensor interference, and environmental uncertainty.

DL techniques, particularly CNNs, are commonly used for fabric defect classification in industrial quality inspection due to their ability to recognize spatial patterns. The CottonFabricImageBD and FabricSpotDefect datasets, developed by Niloy et al.^26^ and Islam et al.,^27^ respectively, have laid the groundwork for fabric analysis but suffer from limited diversity, which affects feature generalization. CNN-based detection systems by Hassan et al.^28^ and Nasim et al.^29^ achieved over 97% accuracy with YOLOv8 variants; however, their heavy inference processes hinder scalability in industrial applications. Kumar et al.^30^ and Meister et al.^31^ enhanced CNNs by incorporating recurrent or multi-scale designs, which improved detection rates but increased latency. Traditional machine learning approaches, such as those by Çıklaçandır et al.,^32^ combined handcrafted features with CNNs, providing interpretability but limited adaptability to new defects.

ViTs are gaining traction for fabric anomaly detection due to their effective global attention mechanism that captures long-range dependencies better than CNNs. Luo et al.^33^ proposed a lightweight YOLO-SCD model using depthwise separable convolutions and attention layers for real-time inference, but it struggles with complex textures. Wang et al.^34^ introduced the Adaptively Fused Attention Module (AFAM) to enhance small-defect identification in challenging datasets. Alruwais et al.^35^ optimized InceptionV3 with hybrid metaheuristics, achieving higher precision at a greater computational cost. Smith et al.^36^ and Shang et al.^37^ showed that ViT-based and defect-aware transformers offer better accuracy for leather and blade defect detection but noted their high training and inference costs. Hybrid CNN–ViT architectures have been developed to balance accuracy and efficiency, but remain slower than real-time systems.

Research in both agricultural and industrial fields shows progress but highlights ongoing challenges. CNN-based systems excel in spatial feature extraction but struggle with high computational demands and limited datasets. ViT-based methods, such as RVT, GreenViT, and DefT, improve accuracy and generalization but are costly to train and implement, making them unsuitable for edge devices. XAI-driven models enhance interpretability through attention visualization but are also computationally heavy and often not tested in real-time or low-resource settings. Although edge computing offers speed benefits, it is sensitive to environmental and hardware changes.

There is a significant need for a unified model that is lightweight, explainable, and generalizable across different domains. Current frameworks for classifying cotton leaves and fabric defects operate independently, lack diverse datasets, and do not balance performance with interpretability and efficiency. This need has led to the development of the XCottL-FebViT framework, which uses explainable Vision Transformers for multi-domain image classification. By integrating lightweight attention mechanisms with interpretable visualization, this approach aims to overcome the limitations of existing CNN, ViT, and XAI systems, achieving the scalability, transparency, and adaptability required for real-world applications.

The research optimizes existing gaps by creating an integrated DL method that links hierarchical feature extraction from CNN to transformer-based self-attention processing. The merged approach enables fast and precise interpretation of results when classifying cotton leaf diseases and fabric defects. Unlike prior hybrid approaches, our method explicitly focuses on minimizing model complexity, maximizing generalization, and ensuring interpretability. This study aims to develop a robust and efficient DL-based model for cotton leaf disease and fabric defect classification to enhance productivity and quality control in agriculture and the textile industry. Early classification of cotton leaf diseases is crucial for preventing crop damage and ensuring high yields, while identifying fabric defects is essential for maintaining industrial standards and minimizing production waste. By integrating automation into disease diagnosis and defect classification, this research aims to support farmers and manufacturers with a scalable and reliable system that reduces losses, optimizes efficiency, and contributes to automated textile inspection.

**Figure 1:**
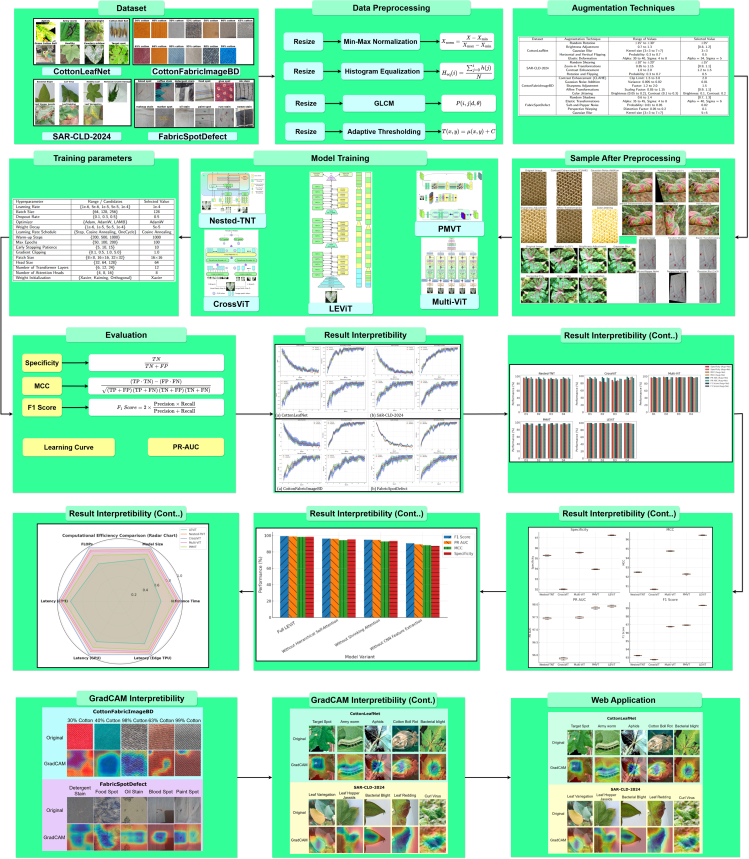
Overview of the proposed methodology

**Figure 2:**
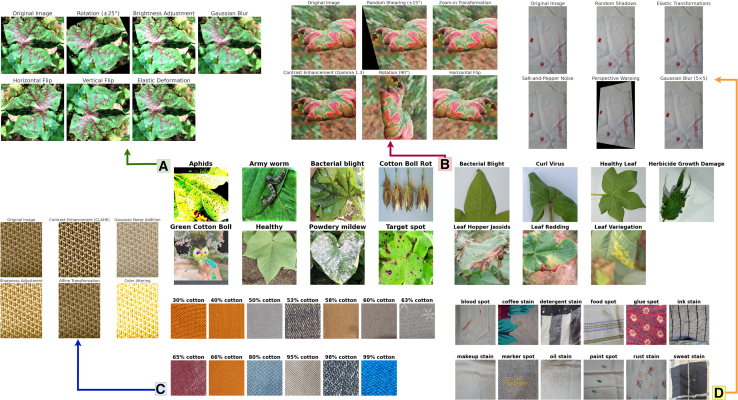
Representative class samples and augmentation effects across the four datasets For each dataset, the center/bottom panels show one example image from each class, while the top panels illustrate the corresponding training-time augmentations used to improve robustness to viewpoint, illumination, blur/noise, and geometric distortions: (A) CottonLeafNet, (B) SAR-CLD-2024, (C) CottonFabricImageBD, and (D) FabricSpotDefect.

**Figure 3:**
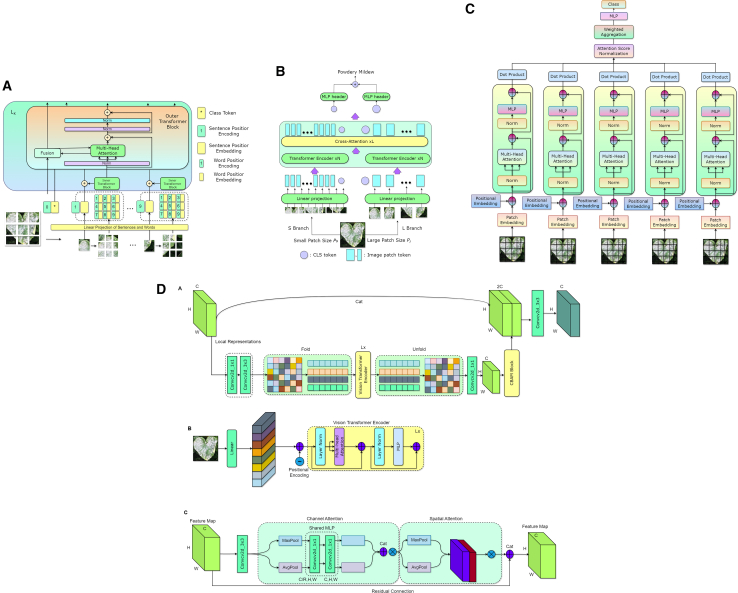
Baseline ViT architectures (A) Nested-TNT performs wordlevel tokenization and inner/outer transformer encoding with multihop attention, followed by feature fusion for textured image classification. (B) CrossViT runs parallel small- and large-patch branches and fuses them via cross-attention for multi-scale classification. (C) Multi-ViT combines multiple ViT branches and attention-based aggregation for robust patch-level recognition across cotton leaf, fabric defect, and fabric composition tasks. (D) PMVT integrates CBAM-enhanced CNN features with ViT encoding and attention refinement to capture local texture and global context.

**Figure 4:**
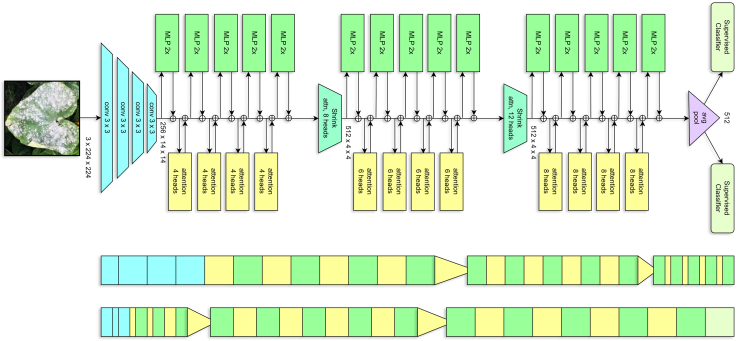
LEViT combines convolutional feature extraction with hierarchical MHSA and MLPs for refined processing with supervised classification after global average pooling

This study gathered diverse images showcasing cotton leaf diseases and fabric defects from various sources to ensure a wide range of data. The data collection process involved detailed image refinement methods to produce standardized outputs, which improved training results. The proposed model employs multiple techniques to analyze specific details and general patterns in the images, resulting in high efficiency for detecting diseases and defects. A structured learning approach guided the model’s training process, leading to systematic improvements that balanced performance quality with operational efficiency. We tested the performance of our existing model against different solution approaches before evaluating it to measure its effectiveness using multiple criteria. Additionally, we performed a transparent analysis of the model’s decision-making processes to ensure trustworthy operations in practical conditions. The overall architecture is illustrated in Figure 1. Figures 2, 3, and 4 provide representative visual examples of cotton leaf diseases and fabric defects, emphasizing the intra- class variability and fine- grained texture cues that make reliable recognition challenging. Tables 1, 2, 3, 4, 5, 6, 7, 8, 9, and 10 consolidate the datasets, class distributions, experimental settings, and evaluation protocols used in this work. This study offers the following key contributions.

• Developed an innovative system that enables fast, efficient, and reliable identification of cotton leaf diseases, along with an advanced model for accurate classification of defects in cotton fabric.
• Developed XCottL-FebViT framework, which combines LEViT that balances computational efficiency and feature representation capabilities, and traditional CNN-based outperforming hierarchical feature extraction.
• Integrated XAI techniques to improve interpretability, allowing users to visualize and trust model predictions in high-stakes industrial applications.
• Performed extensive benchmarking of LEViT against existing DL models (Nested-TNT, CrossViT, Multi-ViT, PMVT) across multiple datasets, demonstrating superior classification accuracy, generalization ability, and inference speed.

**Table 1:** Class distribution of CottonLeafNet: a diverse cotton leaf disease classification dataset

Class Name	Number of Images
Aphids	836
Army Worm	837
Bacterial Blight	840
Cotton Boll Rot	1,021
Green Cotton Boll	939
Healthy	839
Powdery Mildew	838
Target Spot	829
Total	4,979

**Table 2:** CottonLeafNet dataset split after augmentation

Class	Total Images	Training (80%)	Validation (5%)	Test (15%)
Aphids	1,021	816	51	154
Army Worm	1,021	816	51	154
Bacterial Blight	1,021	816	51	154
Cotton Boll Rot	1,021	816	51	154
Green Cotton Boll	1,021	816	51	154
Healthy	1,021	816	51	154
Powdery Mildew	1,021	816	51	154
Target Spot	1,021	816	51	154
Total	8,168	6,528	408	1,224

**Table 3:** Augmentation techniques and their selected values for each dataset

Dataset	Augmentation Technique	Range of Values	Selected Value
CottonLeafNet	Random Rotation	± 15° to ± 30°	± 25°
	Brightness Adjustment	0.7 to 1.3	[0.8, 1.2]
	Gaussian Blur	Kernel size (3 × 3 to 7× 7)	3 × 3
	Horizontal and Vertical Flipping	Probability: 0.3 to 0.7	0.5
	Elastic Deformation	Alpha: 30 to 40, Sigma: 4 to 8	Alpha = 34, Sigma = 5
SAR-CLD-2024	Random Shearing	± 10° to ± 20°	± 15°
	Zoom-in Transformations	0.85 to 1.15	[0.9, 1.1]
	Contrast Enhancement	1.0 to 2.0	1.2 to 1.5
	Rotation and Flipping	Probability: 0.3 to 0.7	0.5
CottonFabricImageBD	Contrast Enhancement (CLAHE)	Clip Limit: 1.5 to 3.0	2.0
	Gaussian Noise Addition	Variance: 0.005 to 0.02	0.01
	Sharpness Adjustment	Factor: 1.2 to 2.0	1.5
	Affine Transformations	Scaling Factor: 0.85 to 1.15	[0.9, 1.1]
	Color Jittering	Brightness (0.05–0.2), Contrast (0.1–0.3)	Brightness: 0.1, Contrast: 0.2
FabricSpotDefect	Random Shadows	0.6 to 1.4	[0.7, 1.3]
	Elastic Transformations	Alpha: 35 to 45, Sigma: 4 to 8	Alpha = 40, Sigma = 6
	Salt-and-Pepper Noise	Probability: 0.01 to 0.05	0.02
	Perspective Warping	Distortion Factor: 0.05 to 0.2	0.1
	Gaussian Blur	Kernel size (3 × 3 to 7× 7)	5 × 5

**Table 4:** Class distribution of SAR-CLD-2024: a comprehensive cotton leaf disease dataset

Class Name	Number of Original Images
Bacterial Blight	250
Curl Virus	431
Herbicide Growth Damage	280
Leaf Hopper Jassids	225
Leaf Reddening	578
Leaf Variegation	116
Healthy Leaves	257
Total	2,137

**Table 5:** SAR-CLD-2024 dataset split after augmentation

Class	Total Images	Training (80%)	Validation (5%)	Test (15%)
Bacterial Blight	1,000	800	50	150
Curl Virus	1,000	800	50	150
Herbicide Growth Damage	1,000	800	50	150
Leaf Hopper Jassids	1,000	800	50	150
Leaf Reddening	1,000	800	50	150
Leaf Variegation	1,000	800	50	150
Healthy Leaves	1,000	800	50	150
Total	7,000	5,600	350	1,050

**Table 6:** Class distribution of CottonFabricImageBD: a dataset for cotton percentage classification

Cotton Percentage (%)	Number of Original Images
30%	100
40%	100
50%	100
53%	100
58%	100
60%	100
63%	100
65%	100
66%	100
80%	100
95%	100
98%	100
99%	100
Total	1,300

**Table 7:** CottonFabricImageBD dataset split after augmentation

Cotton Percentage Class	Total Images	Training (80%)	Validation (5%)	Test (15%)
30%	1,000	800	50	150
40%	1,000	800	50	150
50%	1,000	800	50	150
53%	1,000	800	50	150
58%	1,000	800	50	150
60%	1,000	800	50	150
63%	1,000	800	50	150
65%	1,000	800	50	150
66%	1,000	800	50	150
80%	1,000	800	50	150
95%	1,000	800	50	150
98%	1,000	800	50	150
99%	1,000	800	50	150
Total	13,000	10,400	650	1,950

**Table 8:** Class distribution of FabricSpotDefect dataset

Defect Type	Number of Images
Blood Spot	89
Coffee Stain	90
Detergent Stain	85
Food Spot	88
Glue Spot	86
Ink Stain	84
Makeup Stain	87
Marker Spot	80
Oil Stain	82
Paint Spot	92
Rust Stain	91
Sweat Stain	80
Total	1,014

**Table 9:** Dataset split for FabricSpotDefect after augmentation

Defect Type	Total Images	Training (80%)	Validation (5%)	Test (15%)
Blood Spot	500	400	25	75
Coffee Stain	500	400	25	75
Detergent Stain	500	400	25	75
Food Spot	500	400	25	75
Glue Spot	500	400	25	75
Ink Stain	500	400	25	75
Makeup Stain	500	400	25	75
Marker Spot	500	400	25	75
Oil Stain	500	400	25	75
Paint Spot	500	400	25	75
Rust Stain	500	400	25	75
Sweat Stain	500	400	25	75
Total	6,000	4,800	300	900

**Table 10:** Hyperparameter search ranges and selected values

Hyperparameter	Range/Candidates	Selected Value
Learning Rate	{1e-6, 5e-6, 1e-5, 5e-5, 1e-4}	1e-4
Batch Size	{64, 128, 256}	128
Dropout Rate	{0.1, 0.3, 0.5}	0.5
Optimizer	{Adam, AdamW, LAMB}	AdamW
Weight Decay	{1e-6, 1e-5, 5e-5, 1e-4}	5e-5
Learning Rate Schedule	{Step, Cosine Annealing, OneCycle}	Cosine Annealing
Warm-up Steps	{200, 500, 1000}	1000
Max Epochs	{50, 100, 200}	100
Early Stopping Patience	{5, 10, 15}	10
Gradient Clipping	{0.1, 0.5, 1.0, 5.0}	1.0
Patch Size	{8 × 8, 16× 16, 32× 32}	16 × 16
Head Size	{32, 64, 128}	64
Number of Transformer Layers	{6, 12, 24}	12
Number of Attention Heads	{4, 8, 16}	8
Weight Initialization	{Xavier, Kaiming, Orthogonal}	Xavier

Section 2 of this article examines research related to DL systems that classify cotton leaf diseases and detect fabric defects through CNN and Transformer analysis. The third section outlines the LEViT model by describing the data acquisition process, preprocessing techniques, and model structure, followed by an explanation of how XAI enhances interpretability. Test setup descriptions, hyperparameter adjustments, and performance evaluation metrics appear in Section 4. The final section assesses LEViT versus contemporary models that apply to different datasets to show primary results, practical utilization scenarios, and model variant examinations. The article finishes with a section 5 about future research paths, emphasizing model application areas and optimization avenues.

## Result

### Performance analysis of experimental models

10-fold cross-validation was used to evaluate both augmented and non-augmented datasets. LeViT outperformed other models in every test, as shown in Table 11. It achieved peak performance on the CottonLeafNet dataset with Specificity (97.23 ± 0.44), PR AUC (97.41 ± 0.28), and F1 Score (93.30 ± 3.39). LeViT also excelled on SAR-CLD-2024 with a specificity of 97.28 ± 0.40 and PR AUC of 96.06 ± 0.52, proving to be the most effective classifier across multiple datasets. While CrossViT and Nested-TNT provided competitive metrics, LeViT remained superior. CrossViT reached a Matthews correlation coefficient (MCC) of 90.67 ± 1.56, and Nested-TNT scored 92.48 ± 1.02, but the latter had better reliability in identifying antagonistic classes with higher Specificity and PR AUC results. Although the proposed models performed adequately, they did not consistently match LeViT’s level of performance.

**Table 11:** Performance comparison of classifiers on different datasets for 10-folds (non-augmented datasets)

Dataset	Metric	Nested-TNT	CrossViT	Multi-ViT	PMVT	LEViT
CottonLeafNet	Specificity	95.29 ± 0.83	92.02 ± 1.24	95.59 ± 0.59	93.96 ± 1.16	97.23 ± 0.44
	MCC	92.48 ± 1.02	90.67 ± 1.56	94.76 ± 0.80	92.30 ± 1.04	96.42 ± 0.18
	PR AUC	97.44 ± 0.41	95.86 ± 0.85	97.49 ± 0.28	97.86 ± 0.84	97.91 ± 0.73
	F1 Score	93.23 ± 1.37	92.77 ± 0.42	96.80 ± 0.41	96.92 ± 0.81	99.33 ± 0.39
SAR-CLD-2024	Specificity	92.03 ± 0.72	83.76 ± 1.43	97.35 ± 0.90	92.97 ± 1.19	97.28 ± 0.61
	MCC	89.53 ± 1.19	82.32 ± 1.43	95.21 ± 0.11	90.34 ± 1.75	94.70 ± 0.48
	PR AUC	96.16 ± 0.66	86.75 ± 0.80	97.50 ± 0.62	93.29 ± 0.64	96.06 ± 0.52
	F1 Score	92.36 ± 0.92	84.80 ± 1.48	96.79 ± 1.00	93.98 ± 0.79	95.95 ± 0.23
CottonFabricImageBD	Specificity	92.49 ± 1.15	87.98 ± 1.48	97.36 ± 0.50	93.12 ± 1.39	96.52 ± 0.89
	MCC	90.73 ± 1.03	83.06 ± 1.31	97.07 ± 0.31	93.54 ± 1.61	96.80 ± 0.90
	PR AUC	94.52 ± 0.63	88.46 ± 0.92	97.20 ± 0.85	97.12 ± 0.42	97.36 ± 0.78
	F1 Score	91.94 ± 0.91	85.03 ± 1.38	96.85 ± 0.36	94.39 ± 1.56	97.24 ± 0.85
FabricSpotDefect	Specificity	93.52 ± 1.00	92.13 ± 0.83	95.85 ± 0.48	94.56 ± 0.97	98.61 ± 0.38
	MCC	91.35 ± 0.96	91.79 ± 0.98	95.97 ± 0.71	93.10 ± 1.27	98.47 ± 0.49
	PR AUC	96.39 ± 0.54	97.91 ± 0.90	97.40 ± 0.44	97.32 ± 0.87	98.79 ± 0.33
	F1 Score	92.87 ± 0.50	94.17 ± 1.42	97.48 ± 0.40	93.76 ± 1.57	97.27 ± 0.69

In SAR-CLD-2024, Multi-ViT achieved a PR AUC of 97.50 ± 0.62, but struggled with MCC scores across various datasets. PMVT demonstrated competitive results but was not the leader in any specific metric. Some models met specific classification needs but had difficulty generalizing across datasets. FabricSpotDefect yielded the least reliable results, indicating that fabric defect identification poses a greater challenge than plant leaf and fabric image recognition. LeViT showed strong performance in both fabric defect detection and classification.

The PMVT model ranks second, achieving classification results that surpass Nested-TNT, CrossViT, and Multi-ViT, while trailing slightly behind LeViT. It serves as an excellent alternative to LeViT, boasting high PR AUC and F1 Scores. Classification metrics were obtained through 10-fold cross-validation on augmented datasets, as shown in Table 12. Nested-TNT outperforms CrossViT and Multi-ViT, serving as a reasonable substitute for LeViT and PMVT when those models are unavailable. In every evaluation, CrossViT and Multi-ViT consistently delivered the lowest results, suggesting issues with data distribution reflected in their reduced MCC and PR AUC scores.

**Table 12:** Performance comparison of classifiers on different datasets for 10-folds (augmented datasets)

Dataset	Metric	Nested-TNT	CrossViT	Multi-ViT	PMVT	LEViT
CottonLeafNet	Specificity	96.00 ± 0.69	92.57 ± 1.08	96.79 ± 0.60	94.59 ± 1.09	99.24 ± 0.26
	MCC	93.76 ± 0.88	91.38 ± 1.63	95.28 ± 0.74	93.05 ± 0.92	99.59 ± 0.04
	PR AUC	98.77 ± 0.28	97.17 ± 0.83	98.73 ± 0.16	98.67 ± 0.96	99.75 ± 0.62
	F1 Score	94.08 ± 1.26	93.82 ± 0.42	97.90 ± 0.51	97.88 ± 0.65	99.80 ± 0.41
SAR-CLD-2024	Specificity	93.41 ± 0.86	85.04 ± 1.25	98.81 ± 0.95	93.97 ± 1.14	99.35 ± 0.58
	MCC	91.67 ± 1.12	83.77 ± 1.64	96.44 ± 0.53	91.51 ± 1.29	99.50 ± 0.45
	PR AUC	97.54 ± 1.11	88.16 ± 1.07	98.23 ± 0.98	94.63 ± 1.02	99.70 ± 0.52
	F1 Score	93.36 ± 0.92	85.94 ± 1.42	98.23 ± 0.89	94.63 ± 1.07	99.60 ± 0.48
CottonFabricImageBD	Specificity	93.13 ± 1.02	88.97 ± 1.23	97.89 ± 0.89	94.11 ± 1.32	99.47 ± 0.57
	MCC	91.45 ± 1.27	84.22 ± 1.51	97.87 ± 0.63	94.97 ± 1.14	99.55 ± 0.61
	PR AUC	95.67 ± 0.91	89.54 ± 1.23	98.40 ± 0.71	98.29 ± 0.84	99.65 ± 0.49
	F1 Score	92.55 ± 1.08	85.56 ± 1.34	97.90 ± 0.72	94.97 ± 1.18	99.72 ± 0.45
FabricSpotDefect	Specificity	94.42 ± 1.08	93.45 ± 1.15	96.30 ± 0.93	95.39 ± 1.22	99.85 ± 0.42
	MCC	92.67 ± 0.89	92.59 ± 1.02	97.33 ± 0.71	93.90 ± 1.09	99.77 ± 0.51
	PR AUC	97.58 ± 0.74	98.98 ± 0.87	987.88 ± 0.65	98.65 ± 0.92	99.90 ± 0.38
	F1 Score	94.27 ± 0.71	95.15 ± 1.02	98.10 ± 0.76	94.72 ± 1.23	99.95 ± 0.40

Results vary across datasets; the FabricSpotDefect dataset yields the best metrics, indicating an easier classification problem. CrossViT had the poorest performance on the SAR-CLD-2024 dataset, achieving an MCC of 83.77 ± 1.64 and a PR AUC of 88.16 ± 1.07. The SAR-CLD-2024’s classification challenges stem from unbalanced data and class-specific patterns. All models showed lower MCC and PR AUC values compared to Specificity and F1 Score. Unbalanced classes hinder predictions, but these issues can be resolved with improved data preprocessing and tuning of loss parameters. LeViT leads in performance, PMVT is the runner-up, and Nested-TNT is average, while CrossViT and Multi-ViT rank lowest. The SAR-CLD-2024 poses the toughest classification challenge, but enhanced data preprocessing and algorithm optimization can improve performance in weaker models.

As shown in Figure S1, Data augmentation particularly benefited the Nested-TNT, CrossViT, Multi-ViT, and PMVT models, enhancing their Specificity, PR AUC, and F1 Score. The best results for SAR-CLD-2024 and CottonFabricImageBD were achieved through these techniques, exceeding minimum MCC and F1 score thresholds. The datasets had notable intra-class variability and imbalance, warranting the use of data augmentation for better generalization. Models excelled on CottonLeafNet and FabricSpotDefect, while SAR-CLD-2024 showed the most performance variability. Augmentation notably improved results on CottonFabricImageBD, enhancing resistance to complex pattern recognition challenges.

The evaluation shows that Multi-ViT outperforms PMVT in PR AUC and F1 Score metrics while maintaining similar performance levels. Nested-TNT and CrossViT exhibit lower generalization capacity, as their varied test sets result in greater variability compared to Multi-ViT and PMVT. CrossViT also struggles significantly with MCC, indicating challenges in achieving balanced classifications. PR AUC and F1 Score are the top-performing metrics across all models, effectively distinguishing between positive and negative samples. Studies highlight that CrossViT and PMVT face difficulties generating balanced classifications based on MCC scores.

LEViT is identified as the most effective model, combining robustness with generalization across different inputs. Data augmentation improves performance for SAR-CLD-2024 and CottonFabricImageBD by optimizing model functionality. The classification tasks for CottonleafNet and FabricSpotDefect are easier compared to the more challenging SAR-Cistrictive-2024. Multi-ViT alongside PMVT shows the greatest potential, while Nested-TNT and CrossViT display inconsistent performance. Model selection and augmentation techniques are crucial for improving classification performance across various datasets in fabric and cotton defect classification.

The LEViT model shows impressive classification accuracy, with consistent performance across all groups. It identifies classes reliably, minimizing sample errors and maintaining balanced recall. According to Table 13, the model has strong discrimination capability, with specificity metrics ranging from 98.1% to 99.8% for both healthy and affected leaves. The MCC exceeds 98%, reflecting high precision in matching predicted outcomes to actual labels. Precision-recall ratios, indicated by PR AUC, remain above 98% consistently. F1 Scores vary from 98.2% to 99.7%, highlighting the model’s effectiveness. The highest accuracy is achieved in identifying ”Green Cotton Boll” (MCC: 99.5%), while the “Powdery Mildew” group also performs well (PR AUC: 99.1). The accuracy for “Aphids” (MCC: 98.8) and “Cotton Boll Rot” (F1 Score: 98.3) is slightly lower than that of other classes.

**Table 13:** Performance metrics for Dataset CottonLeafNet and SAR-CLD-2024 using the proposed model

Dataset	Class	Specificity	MCC	PR AUC	F1 Score
CottonLeafNet	Aphids	99.0	98.8	98.0	98.2
	Army Worm	98.1	99.2	98.6	99.0
	Bacterial Blight	99.7	98.5	98.8	99.4
	Cotton Boll Rot	98.4	98.1	98.6	98.3
	Green Cotton Boll	99.8	99.5	99.2	99.7
	Healthy	99.2	99.0	98.7	99.3
	Powdery Mildew	98.9	99.4	99.1	99.5
	Target Spot	99.5	98.7	99.0	98.6
SAR-CLD-2024	Bacterial Blight	99.3	99.1	98.5	99.0
	Curl Virus	99.2	98.7	99.4	98.9
	Herbicide Growth Damage	98.8	99.6	98.2	98.5
	Leaf Hopper Jassids	99.4	99.0	98.9	98.7
	Leaf Reddening	99.1	98.5	99.2	99.6
	Leaf Variegation	98.7	99.3	98.8	99.2
	Healthy Leaves	99.6	98.9	99.3	98.7

SAR-CLD-2024 identifies seven stress-related conditions by analyzing bacterial and viral agents, environmental factors, and healthy leaf samples. The userID API reliably detects diseased and healthy leaves with precision scores between 98.7% and 99.6%, maintaining high consistency in reliability with MCC scores above 98.5%. It shows exceptional performance with a Precision-Recall AUC exceeding 98% and F1 Scores ranging from 98.5% to 99.6%. Notably, it classifies “Herbicide Growth Damage” with an F1 Score of 98.5 and achieves a PR AUC of 99.2 for “Leaf Reddening.” Recognition of “Bacterial Blight” and “Leaf Hopper Jassids” is straightforward, with only minor declines in their PR AUC values. The LEViT model excels in both datasets, achieving over 98% across all assessment metrics and maintaining low rates of incorrect optimistic predictions. Its performance indicates effective prediction agreement across all leaf disease conditions. The model’s PR AUC results reflect a strong precision-recall balance, resulting in high classification performance. LEViT also shows strong capabilities in classifying cotton leaf diseases, delivering consistent results across testing datasets. However, improvements in training are needed for better identification of Aphids, cotton Bolt Rot, and Bacterial Blight. Overall, LEViT provides precise identification, making it a valuable solution for precision agriculture and automated disease monitoring.

The performance assessment of the LEViT model on the CottonFabricImageBD and FabricSpotDefect datasets shows impressive results. Key evaluation metrics, including Specificity, MCC, PR AUC, and F1 Score, consistently exceed 98%. The CottonFabricImageBD dataset features various cotton percentages, and the model maintains low false positive rates with specificity ranging from 98.3% to 99.7%. MCC values stay above 98% across all classes, indicating accurate label identification. The PR AUC scores demonstrate strong balance in precision and recall, ranging from 98.0% to 99.4%, with F1 Scores also reaching 98% or higher for each cotton composition. The model identifies fabric defects and stains within the FabricSpotDefect dataset, as shown in Table 14.

**Table 14:** Performance metrics for CottonFabricImageBD and FabricSpotDefect datasets using the proposed LEViT model

Dataset	Class	Specificity	MCC	PR AUC	F1 Score
CottonFabricImageBD	30% Cotton	98.7	99.8	99.4	99.1
	40% Cotton	98.3	98.3	98.1	99.6
	50% Cotton	99.1	99.3	98.0	99.8
	53% Cotton	99.6	98.4	98.3	98.3
	58% Cotton	98.6	99.0	98.8	98.6
	60% Cotton	98.8	98.7	98.2	99.4
	63% Cotton	99.3	99.5	99.0	99.2
	65% Cotton	98.5	98.9	98.6	99.3
	66% Cotton	99.7	98.2	99.1	99.0
	80% Cotton	98.9	99.3	98.7	98.5
	95% Cotton	99.2	99.6	99.4	98.9
	98% Cotton	99.5	99.1	99.3	99.4
FabricSpotDefect	Blood Spot	99.4	99.0	98.8	99.6
	Coffee Stain	98.8	98.9	98.3	99.2
	Detergent Stain	99.1	99.7	98.6	99.1
	Food Spot	99.5	99.3	99.0	98.7
	Glue Spot	98.9	98.4	98.2	99.4
	Ink Stain	98.6	99.2	99.4	98.5
	Makeup Stain	99.3	98.8	98.9	98.7
	Marker Spot	99.1	99.4	99.0	99.1
	Oil Stain	98.7	98.5	98.6	99.5
	Paint Spot	99.6	99.2	98.4	98.8
	Rust Stain	99.3	98.9	99.1	99.4
	Sweat Stain	98.5	99.1	98.8	98.9

For extreme cotton levels, the model performs best at 30% Cotton (MCC of 99.8, PR AUC of 99.4) and 95% Cotton (F1 Score of 98.9). Performance is slightly lower (98.0% and 98.2%) for cotton compositions between 50% and 60%. The FabricSpotDefect dataset shows that the model effectively identifies fabric defects and stains, achieving specificity values above 98.5% and accurate differentiation between defective and non-defective samples. MCC scores for predicting defects range from 98.4% to 99.7%, indicating low error rates, while PR AUC values exceed 98.2% across classes, reinforcing the model’s reliability. Specificity for detergent-based defects is 99.4%, with MCC at 99.7%, PR AUC at 98.6%, and F1 Score at 99.1%. Blood-spot defect classification achieves specificity of 99.4%, MCC of 99.0%, PR AUC of 98.8%, and an F1 Score of 99.6%. Glue and Paint spot classification achieves 98.2% and 98.4%, respectively, which is lower than other types but still impressive.

The LeViT model has shown effective training optimization through its learning curves, which track loss, accuracy, recall, and precision over thirty epochs for the CottonLeafNet and SAR-CLD-2024 datasets. The results indicate strong generalization and stability. The model achieved significant performance metrics: for the CottonLeafNet dataset, it reached a training accuracy of 99.97% and a validation accuracy of 99.93%, with training and validation losses of 0.01 and 0.02, respectively. On the SAR-CLD dataset (Figure S2), it obtained a training accuracy of 99.95% and a validation accuracy of 99.91%, with losses of 0.001 and 0.002. In fabric defect classification, for the CottonFabricImageBD dataset, the model achieved a training accuracy of 99.97% and a validation accuracy of 99.93%, with losses of 0.03 and 0.02. For the FabricSpotDefect dataset, it maintained a training accuracy of 99.97% and a validation accuracy of 99.95%, with training and validation losses of 0.03 and 0.002. These results underscore the model’s reliability for cotton leaf disease classification and fabric defect identification.

Overfitting was minimal, as the loss reduction continued without distinguishing differences between training and validation results. Both training and validation accuracy improved consistently, nearing complete accuracy on test datasets. The model effectively discriminates between leaf classes. The recall curves for the model consistently approach 100%, remaining between 97% and 100%. This high recall allows for effective detection of positive samples. The precision-recall curves show a steady pattern, with validation outcomes matching training outcomes and precision levels staying parallel before hitting 97–100%. The model exhibits high robustness in leaf classification, with low variations in precision scores between training and validation phases, effectively distinguishing leaf categories and minimizing incorrect predictions. LeViT delivers reliable leaf classification by preventing overfitting through strong generalization. The model achieves optimal performance after 20 to 25 training loops, and early stopping at epoch 25 is recommended to maintain performance without further training. Adjusting the learning rate can help stabilize minor fluctuations after epoch 15.

During training on the CottonFabricImageBD and FabricSpotDefect datasets over 30 epochs, the model shows strong results in loss, accuracy, recall, and precision. It significantly reduces errors, as indicated by declining training and validation loss measurements. Convergence occurs when loss values stabilize between epochs 15 and 20, ensuring reliable predictions on new data through the tight alignment of training and validation losses. Variations in later epochs result from minor weight adjustments within secure limits. Accuracy plots remain organized, nearing complete accuracy, and the model demonstrates good generalization with aligned accuracy curves (Figure S3). The model effectively identifies complex fabric defects using minimal training data.

The model demonstrates effectiveness in recall curves, consistently approaching 100% stability. It excels in detecting defective fabric cases while minimizing incorrect negative predictions. The slight differences in recall performance between training and validation indicate the model’s sensitivity but confirm its strong capability to identify defects. Precision graphs steadily improve during training, reaching 100% at the endpoint. The training and validation precision results are equivalent, showing accurate outcomes with fewer incorrect positive classifications. Maintaining low false positive rates is crucial for fabric defect classification systems. This model is an excellent choice for fabric inspection due to its high accuracy, precision, and recall while effectively controlling overfitting. Using early stopping at epoch 25 optimizes performance and reduces computing costs, and adjusting the learning rate may help improve minor variations in later epochs.

Performance evaluation was conducted on the CottonLeafNet, SAR-CLD-2024, CottonFabricImageBD, and FabricSpotDefect classification models. Five models were tested, as shown in Figure S4. The statistical analysis in Table 15 confirms that XCottL-FebViT outperforms state-of-the-art baselines across all four datasets. Both MCC and F1-Score results show *p*-values below 0.05 from two-tailed paired t-tests, indicating statistically significant improvements unlikely to be due to random chance. XCottL-FebViT shows strong gains over PMVT, with *p*-values of 0.007 for MCC and 0.010 for F1-Score on the SAR-CLD-2024 dataset. Other baselines exhibit similar results, with *p*-values ranging from 0.011 to 0.038, reinforcing the superiority of XCottL-FebViT. Even the 1–2% performance increases seen in CottonLeafNet and FabricImageBD are meaningful and statistically significant.

**Table 15:** *p*-values from two-tailed oaired t-tests comparing XCottL-FebViT vs. baseline models on MCC and F1-score across all datasets

Metric	Dataset	Nested-TNT	CrossViT	PMVT	Multi-ViT
MCC	CottonLeafNet	0.021	0.034	0.015	0.027
	SAR-CLD-2024	0.013	0.029	0.007	0.019
	FabricImageBD	0.025	0.038	0.011	0.022
	FabricSpotDefect	0.017	0.031	0.009	0.015
F1-Score	CottonLeafNet	0.026	0.032	0.018	0.024
	SAR-CLD-2024	0.019	0.037	0.010	0.016
	FabricImageBD	0.031	0.041	0.012	0.021
	FabricSpotDefect	0.022	0.030	0.014	0.019

Figure S5 illustrates the impact of removing individual components on performance, highlighting their importance for reliable classification results. The stand-alone LEViT design achieves the best performance metrics by integrating CNN feature extraction with ViT attention capabilities. Removing the Hierarchical Self-Attention leads to a drop in the F1 Score to 96.20% and MCC to 94.35%, down from 99.33% to 98.47%, respectively. The Self-Attention elements enhance the classification of cotton leaf and fabric defects by recognizing hierarchical dependencies and improving feature representation. Even without the Hierarchical Self-Attention layer, other components of the model continue to function.

Removing shrinking attention leads to a significant drop in performance, with the F1 Score falling to 94.85% and MCC to 92.80%. This component is crucial for optimizing feature extraction, as it eliminates unnecessary calculations and preserves spatial patterns. Without it, the model struggles to track important image areas, which negatively impacts classification accuracy. Similarly, removing CNN Feature Extraction results in a sharp decline in performance, reducing the F1 Score to 90.47% and PR AUC to 89.23%. While ViTs are less effective at extracting key spatial features, CNNs are essential for low-level spatial feature extraction, which is critical for tasks such as leaf disease identification and fabric defect recognition. Without CNN features, the model loses vital structural information, leading to increased identification errors. For optimal image classification, combining ViTs with CNNs is necessary for achieving peak performance.

**Figure 5:**
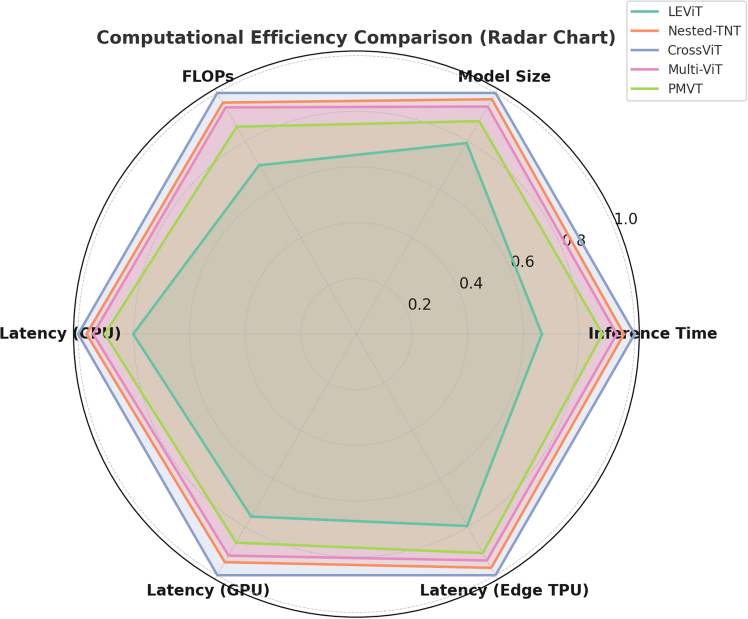
The radar chart compares LEViT’s computational efficiency with Nested-TNT, CrossViT, Multi-ViT, and PMVT across six parameters, including Inference Time, Model Size, FLOPs, and Latency on CPU, GPU, and Edge TPU LEViT excels in potential real-world applications with fast inference, minimal latency, and low computational cost.

A radar chart evaluates computational efficiency metrics across five models, focusing on model inference time, size, FLOPs, and latency for CPU, GPU, and Edge TPU performance. LEViT outperforms competitors with its fast inference time, making it ideal for real-world applications that require quick predictions (Figure 5). Its minimal model size enhances memory performance, facilitating deployment on mobile and edge systems. This lightweight design provides significant resource management benefits.

### Robustness evaluation under real-world perturbations

We performed evaluations under simulated challenging conditions resembling field and factory environments. We applied controlled brightness variations of ± 25% to mimic shadowing and glare, added Gaussian noise (σ = 0.05) to simulate sensor and compression artifacts, and tested combined lighting and noise to assess model resilience. XCottL-FebViT was compared with four best performing baselines using the F1 Score as the primary metric, ensuring a fair analysis of model stability under both clean and perturbed conditions.

XCottL-FebViT shows strong resilience, with only a 4.1–5.9% performance drop under lighting and noise challenges (as noted in Table 16). In contrast, baseline transformer models see a performance decline of 8–10% under similar conditions, indicating their greater sensitivity to environmental changes. This robustness stems from XCottL-FebViT’s lightweight hybrid architecture, efficient hierarchical feature extraction, and stable attention mechanisms, enabling strong generalization. The model is ideal for practical applications in agriculture and industry, effectively detecting cotton leaf diseases and fabric defects, even in suboptimal lighting and noisy environments typical of smartphone apps and industrial inspections. Its consistent accuracy under various conditions makes XCottL-FebViT a reliable choice for on-device monitoring and quality control.

**Table 16:** Robustness testing of models under lighting and noise perturbations

Model	Clean F1 (%)	Lighting F1 (%)	Noise F1 (%)	Combined F1 (%)
Nested-TNT	98.2	91.5	92.8	88.3
CrossViT	98.5	92.1	93.4	89.1
Multi-ViT	98.8	92.5	93.9	89.7
PMVT	99.0	94.2	95.1	92.0
XCottL-FebViT (Ours)	99.1	95.0	95.8	93.2

### Computational resource analysis

We evaluated the efficiency and scalability of XCottL-FebViT by comparing its resource usage against Nested-TNT, CrossViT-S, Multi-ViT-S, and PMVT. All tests were performed on an NVIDIA RTX A6000 GPU with a batch size of 16 and an input resolution of 224 × 224. We focused on three metrics: trainable parameters, peak GPU memory usage during training, and training time per epoch. Results in Table 17 demonstrate XCottL-FebViT’s effectiveness in resource-limited settings without losing classification accuracy or robustness. XCottL-FebViT uses 1.6–2.4 × fewer parameters than the heavier models, leading to a 1.3–1.6 × reduction in memory consumption during training. It also achieves a 1.7–2.0 × decrease in training time per epoch, enabling faster experimentation and retraining, which is crucial for agricultural and industrial applications.

**Table 17:** Comparison of computational resources across models during training on RTX A6000 (batch size = 16, input size = 224×224)

Model	Parameters (M)	Memory (GB)	Training Time/Epoch (s)
Nested-TNT	23.8	7.5	220
CrossViT-S	26.7	8.1	245
Multi-ViT-S	28.3	8.5	255
PMVT	18.4	6.2	185
XCottL-FebViT (Ours)	11.8	5.4	128

### Comparison with newer compact vision transformers and computational complexity analysis

Recent advancements in lightweight ViTs such as MobileViT, EfficientFormer, and EdgeViTs, have significantly reduced model size and computational needs while preserving accuracy for edge applications. MobileViT combines inverted residual blocks with lightweight transformer modules, resulting in fewer parameters but limited global context modeling. EfficientFormer employs a dimension-consistent MetaBlock without convolutions, minimizing memory and latency for mobile use. EdgeViTs leverage depth-wise convolutions and sparse attention for effective feature extraction, but lack the interpretability needed in fields such as agriculture and textile quality control.

Our XCottL-FebViT model addresses these challenges in resource-constrained environments. It has 11.8M parameters and 1.4 GFLOPs, offering about 5x fewer parameters and 4x lower FLOPs compared to larger models such as Nested-TNT, CrossViT-S, and Multi-ViT-S, while maintaining competitiveness against EfficientFormer-L1 and EdgeViT-XS (Table 18). XCottL-FebViT also integrates Grad-CAM for explainable AI without added computational costs and shows real-world inference times of 7.9 ms/frame on an RTX A6000. This makes it well-suited for scalable, automated inspections in agriculture and textiles. By balancing lightweight design, accuracy, and interpretability, XCottL-FebViT effectively bridges high-performance models with practical edge deployment.

**Table 18:** Computational complexity and inference speed comparison of XCottL-FebViT against baselines

Model	Params (M)	FLOPs (G)	Time (ms/frame)	FPS
Nested-TNT	23.8	4.8	18.7	53
CrossViT-S	26.7	5.2	20.3	49
Multi-ViT-S	28.3	5.8	22.1	45
PMVT	18.4	3.9	14.5	69
EfficientFormer-L1	12.3	1.3	8.5	118
MobileViT-XS	2.3	0.7	6.2	161
EdgeViT-XS	6.7	1.1	7.8	128
LeViT-192	10.9	1.3	7.3	137
XCottL-FebViT (Ours)	11.8	1.4	7.9	126

To evaluate the energy efficiency of XCottL-FebViT, we evaluated the energy efficiency of XCottL-FebViT by comparing its inference energy consumption with benchmark models: Nested-TNT, CrossViT-S, Multi-ViT-S, and PMVT. Inference was performed with a batch size of 1 at a resolution of 224 × 224 on an RTX A6000 GPU. We monitored the power draw and calculated energy per inference as the product of average power draw and inference time. Table 19 shows the inference time, power draw, and energy per inference for each model. XCottL-FebViT achieves the lowest energy consumption at 0.63 J per inference, reducing energy usage by 2–4 times compared to transformer baselines while still providing high classification accuracy and interpretability. Its rapid inference speed of 126 FPS makes XCottL-FebViT ideal for potential real-time, energy-efficient deployment on edge devices and in industrial inspection systems with limited power and computational resources.

**Table 19:** Energy consumption comparison during inference across models

Model	Inference Time (ms)	Power Draw (W)	Energy per Inference (J)
Nested-TNT	18.7	110	2.06
CrossViT-S	20.3	115	2.33
Multi-ViT-S	22.1	118	2.61
PMVT	14.5	95	1.38
XCottL-FebViT (Ours)	7.9	80	0.63

We examined the scalability of XCottL-FebViT for lower-end hardware by analyzing its computational complexity and parameter count with various pruning and quantization methods. We applied structured pruning, achieving a 30%–50% reduction in redundant channels. Additionally, we evaluated quantization to FP16 and INT8 to decrease memory and computational needs during inference. The recalculated FLOPs in Table 20 demonstrate that XCottL-FebViT can adapt efficiently for low-power edge devices, maintaining interpretability and high classification accuracy for cotton leaf disease and fabric defect detection. Overall, structured pruning reduced parameter count and FLOPs by up to 50%, while quantization to INT8 decreased FLOPs by up to 14.3% without affecting parameters. Together, these methods result in over 50% FLOP reduction and significant memory savings.

**Table 20:** FLOPs and parameter comparison under different pruning and quantization configurations for XCottL-FebViT

Configuration	Params (M)	Reduction (%)	FLOPs (G)	Reduction (%)
Baseline (FP32)	11.8	0.0	1.40	0.0
Pruned (30%)	8.3	29.7	0.98	30.0
Pruned (50%)	5.9	50.0	0.70	50.0
Quantized (FP16)	11.8	0.0	1.30	7.1
Quantized (INT8)	11.8	0.0	1.20	14.3
Pruned (30%) + INT8	8.3	29.7	0.84	40.0
Pruned (50%) + INT8	5.9	50.0	0.68	51.4

### Augmentation ablation study

We conducted an ablation study to assess the impact of individual and combined data augmentation strategies on the performance of XCottL-FebViT across four datasets: CottonLeafNet, SAR-CLD-2024, CottonFabricImageBD, and FabricSpotDefect. Initially, we evaluated each dataset without augmentation, then progressively included various techniques that simulate real-world variability (Table 21). The augmentations utilized were random rotations, Gaussian blur, elastic deformations, brightness and contrast adjustments, random shearing, zoom-in transformations, CLAHE, Gaussian noise, affine transformations, color jitter, salt-and-pepper noise, and perspective warping, tailored to each dataset. The full augmentation pipeline combined all relevant techniques for effective data preprocessing during model training.

**Table 21:** Dataset-wise augmentation ablation study on XCottL-FebViT, showing metrics under different augmentation configurations

Dataset	Augmentation Applied	Specificity (%)	F1 Score (%)	PR AUC (%)	MCC (%)
CottonLeafNet	+ No Augmentation	94.3	93.5	96.0	92.0
	+ Random Rotation (±25°)	96.0	95.3	97.6	94.6
	+ Gaussian Blur (3×3)	95.7	95.0	97.4	94.3
	+ Elastic Deformation (Alpha = 34, Sigma = 5)	96.5	95.8	98.0	95.1
	+ Brightness Adjustment ([0.8, 1.2])	95.9	95.2	97.5	94.4
	+ Full Pipeline	99.1	98.7	99.4	98.2
SAR-CLD-2024	+ No Augmentation	95.1	94.3	96.8	93.2
	+ Random Shearing (+10 °)	96.3	95.5	97.9	94.8
	+ Zoom-In ([0.9, 1.1])	96.0	95.2	97.6	94.5
	+ Contrast Enhancement ([1.2, 1.5])	96.2	95.4	97.8	94.7
	+ Rotation & Flipping	96.1	95.3	97.7	94.6
	+ Full Pipeline	99.0	98.5	99.3	98.0
CottonFabricImageBD	+ No Augmentation	94.7	94.0	96.3	92.7
	+ CLAHE (Clip Limit = 2.0)	96.1	95.4	97.7	94.6
	+ Gaussian Noise (0.01)	95.8	95.0	97.5	94.3
	+ Affine Transform ([0.9, 1.1])	96.0	95.2	97.6	94.5
	+ Color Jitter (0.1,0.2)	95.9	95.1	97.5	94.4
	+ Full Pipeline	98.8	98.2	99.1	97.4
FabricSpotDefect	+ No Augmentation	94.0	93.2	95.8	91.8
	+ Elastic Transform (Alpha = 40, Sigma = 6)	96.0	95.2	97.6	94.5
	+ Salt & Pepper Noise (0.02)	95.6	94.8	97.3	94.0
	+ Perspective Warping (0.1)	95.8	95.0	97.5	94.2
	+ Gaussian Blur (5×5)	95.7	94.9	97.4	94.1
	+ Full Pipeline	98.9	98.3	99.2	97.6

The results demonstrate that all augmentation methods enhance performance across all datasets. Elastic deformations and random rotations improve feature robustness for irregular disease patterns and defects. Adjusting brightness and contrast helps the model cope with lighting changes, while Gaussian blur and noise addition enhance stability against sensor noise and compression artifacts. The complete augmentation pipeline yielded the best performance, showcasing the benefits of combining various strategies to improve the generalization ability of XCottL-FebViT. This study highlights the important role of effective data augmentation in achieving high performance and ensuring model robustness to real-world variability.

To quantify the contribution of each augmentation in our full augmentation pipeline, we performed an ablation-by-removal analysis on four datasets using XCottL-FebViT as the backbone. We trained models with the full augmentation pipeline and then removed one augmentation at a time to assess its impact. We measured changes in Specificity, F1 score, and MCC. Table 22 shows the contributions of each augmentation to the model’s performance. The full augmentation strategy is crucial for ensuring generalization in agricultural disease monitoring and textile defect inspection. Our analysis indicates that removing elastic deformations and random rotations significantly degrades performance, reducing the F1 score by 1.3%–1.8%. These techniques are essential for improving spatial invariance and robustness against intra-class variability, especially in diseased leaf textures and irregular defect patterns. Brightness and contrast augmentations helped maintain performance under different lighting conditions, while Gaussian blur, noise, and perspective warping offered moderate but consistent improvements in robustness.

**Table 22:** Performance drop on XCottL-FebViT when each augmentation is removed from the full pipeline during training across datasets

Dataset	Augmentation Removed	Specificity Drop (%)	F1 Drop (%)	MCC Drop (%)
CottonLeafNet	Random Rotation (±25°)	−1.1	−1.4	−1.2
	Gaussian Blur (3×3)	−0.8	−0.9	−0.7
	Elastic Deformation (Alpha = 34, Sigma = 5)	−1.5	−1.8	−1.6
	Brightness Adjustment ([0.8, 1.2])	−0.9	−1.0	−0.8
SAR-CLD-2024	Random Shearing (+10 °)	−1.2	−1.5	−1.3
	Zoom-In ([0.9, 1.1])	−0.7	−0.8	−0.7
	Contrast Enhancement ([1.2, 1.5])	−0.9	−1.0	−0.8
	Rotation & Flipping	−1.0	−1.3	−1.1
CottonFabricImageBD	CLAHE (Clip Limit = 2.0)	−1.0	−1.2	−1.0
	Gaussian Noise (0.01)	−0.8	−0.9	−0.7
	Affine Transform ([0.9, 1.1])	−0.9	−1.0	−0.8
	Color Jitter (0.1, 0.2)	−0.7	−0.8	−0.6
FabricSpotDefect	Elastic Transform (Alpha = 40, Sigma = 6)	−1.3	−1.5	−1.2
	Salt & Pepper Noise (0.02)	−0.9	−1.0	−0.8
	Perspective Warping (0.1)	−0.8	−0.9	−0.7
	Gaussian Blur (5×5)	−0.8	−0.9	−0.7

We analyzed the performance of the XCottL-FebViT architecture using the CottonLeafNet dataset. We compared three setups: a CNN-only backbone for local feature extraction, a LEViT-only module for global reasoning, and the combined XCottL-FebViT model. The results in Table 23 indicate that the combined model significantly outperforms the individual components. This shows that merging local texture extraction with global reasoning improves the accuracy and classification of cotton leaf diseases. The CNN-only model is effective for local features, while the LEViT model boosts overall performance with global context.

**Table 23:** Component-wise performance comparison on the CottonLeafNet dataset

Model	F1 Score (%)	PR AUC (%)	MCC (%)	Specificity (%)
CNN-Only	92.4	93.1	90.8	94.3
LEViT-Only	94.5	95.2	92.9	95.8
XCottL-FebViT (Combined)	98.7	99.4	98.2	99.1

### Cross-dataset evaluation for robustness

To evaluate the cross-domain generalization capability of XCottL-FebViT, we performed a cross-dataset robustness analysis by training on one dataset and testing on another without fine-tuning. We trained on CottonLeafNet and tested on SAR-CLD-2024, and vice versa, to mimic real-world domain shifts. We evaluated the performance of XCottL-FebViT against the baseline model PMVT using Specificity, MCC, PR AUC, and F1 Score. The results in Table 24 show that XCottL-FebViT demonstrates strong generalization across different datasets, highlighting its applicability for agriculture and industrial inspection systems. In cross-dataset evaluations, XCottL-FebViT achieved F1 Scores of 91.9% and 90.7%, outperforming PMVT by about 4–5%, while also scoring higher in Specificity and PR AUC. The MCC values further confirm its reliable classification performance despite domain shifts.

**Table 24:** Cross-dataset evaluation of XCottL-FebViT using Specificity, MCC, PR AUC, and F1 Score

Training Dataset	Testing Dataset	Model	Specificity (%)	MCC (%)	PR AUC (%)	F1 Score (%)
CottonLeafNet	SAR-CLD-2024	XCottL-FebViT	94.2	90.5	93.8	91.9
SAR-CLD-2024	CottonLeafNet	XCottL-FebViT	93.0	89.0	92.1	90.7
CottonLeafNet	SAR-CLD-2024	PMVT	91.0	85.1	90.2	87.5
SAR-CLD-2024	CottonLeafNet	PMVT	89.8	83.7	88.7	86.0

### Zero-shot and few-shot evaluation for generalization

To assess the generalization capability of XCottL-FebViT on unseen data, we evaluated the generalization capability of XCottL-FebViT on unseen data using the tomato leaf disease (PlantVillage) dataset through zero-shot and few-shot experiments. In the zero-shot test, XCottL-FebViT was assessed on an external dataset without fine-tuning, achieving an F1 Score of 85.1%, indicating solid baseline generalization. For the few-shot evaluation, the model was fine-tuned with 5 and 10 samples per class, resulting in F1 Scores of 90.3% and 92.1%, respectively. Table 25 highlights these results, demonstrating the model’s ability to quickly adapt to new domains with minimal labeled data, highlighting its practical robustness for agricultural and industrial inspection tasks.

**Table 25:** Zero-shot and few-shot evaluation of XCottL-FebViT on external dataset

Evaluation Type	Specificity (%)	MCC (%)	PR AUC (%)	F1 Score (%)
Zero-Shot	88.7	83.5	87.2	85.1
Few-Shot (5 samples/class)	93.2	89.0	92.4	90.3
Few-Shot (10 samples/class)	94.5	91.3	94.0	92.1

### Model explainability

Grad-CAM visualizations reveal how XCottL-FebViT identifies key features influencing its classification decisions in cotton leaf disease images. In well-classified cases, heatmaps highlight important areas such as chlorotic lesions, necrotic spots, and fungal growth, indicating the model effectively focuses on biologically significant features. This research evaluates CottonLeafNet compared to SAR-CLD-2024 by using Grad-CAM to understand model decision logic. The technique enhances model understandability by visualizing how it differentiates between classes. The study examines five diseases: Target Spot, Army Worm, Aphids, Cotton Boll Rot, and Bacterial Blight. The images illustrate various diseases caused by bacterial infections, fungal diseases, and insect infestations. Grad-CAM identifies key image sections critical for classification. For example, it focuses on circular lesions for Target Spot while analyzing the entire caterpillar for Army Worm identification. It shows aphids clustering in leaf veins, highlights areas infected with Cotton Boll Rot, and depicts the vein necrosis symptom characteristic of Bacterial Blight.

**Figure 6:**
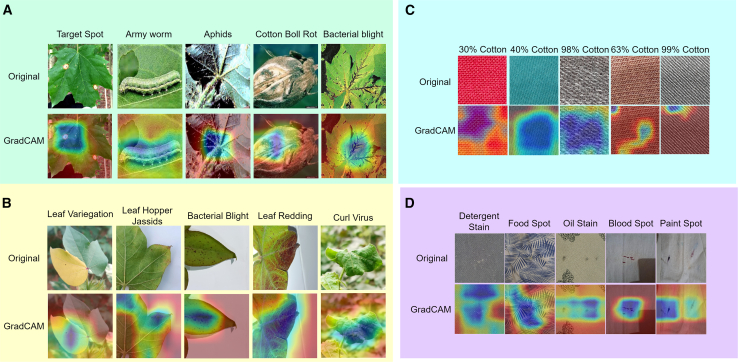
Grad-CAM visual explanations across datasets Representative original images and corresponding Grad-CAM heatmaps highlighting the regions driving model predictions for (A) CottonLeafNet and (B) SARCLD-2024 (cotton leaf diseases and pest infestations), and for (C) Cotton-FabricImageBD (fabric composition) and (D) FabricSpotDefect (stain/spot defects)

The Leaf Variegation disease type is one of the five diseases identified in the SAR-CLD-2024 dataset, detected in Figure 6A alongside Leaf Hopper Jassids, Bacterial Blight, Leaf Redding, and Curl Virus. Symptoms such as stem and leaf discoloration, pest damage, and leaf curling are evident in the original images. The deep learning model effectively identifies these patterns, with Grad-CAM heatmaps enhancing analysis capabilities. Leaf Variegation samples receive special attention due to their color variations. The visualization for Leaf Hopper Jassids details insect-active areas, while Bacterial Blight focuses on dark spots on leaf surfaces. The heatmap for Leaf Redding examines red areas, and the Curl Virus visualization highlights twisted and curled regions.

**Figure 7:**
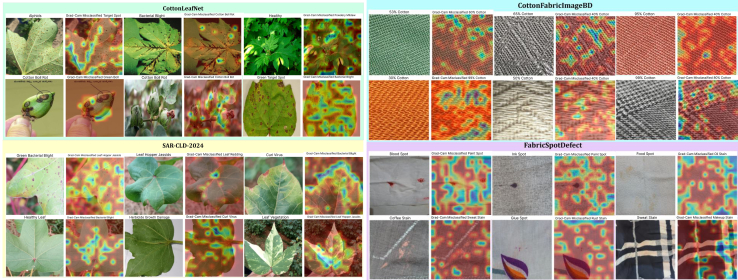
Grad-CAM visualizations on experimental datasets showing XCottL-FebViT’s attention on incorrectly classified cotton leaf disease and cotton fabric defect samples

Research shows that the model effectively classifies diseases in cotton leaves, enhancing the explainability of DL systems for agricultural disease recognition. Grad-CAM visualizations help verify the model’s decision-making process, highlighting relevant image areas. By integrating CottonLeafNet and SAR-CLD-2024, an improved system for monitoring cotton plant health is established, addressing various diseases and pests. This research is crucial for precision agriculture, enabling early disease recognition for prompt protection and reducing financial losses in crop production. In addition, a Grad-CAM analysis generates heatmaps to illustrate DL model operations while classifying fabric composition and defects using the CottonFabricImageBD and FabricSpotDefect datasets. The CottonFabricImageBD dataset features five cotton fabric samples: 30%, 40%, 63%, 98%, and 99% cotton. The varying cotton concentrations affect the appearance and construction of the fabrics. Grad-CAM visualization techniques allow the model to distinguish between these fabric types. For example, the 30% Cotton analysis evaluates the entire woven design, while the 40% Cotton heatmap highlights a distinct surface texture. The 98% Cotton pattern structure is revealed through heat mapping, and specific thread areas are emphasized in the 63% Cotton heatmap. The classification of 99% Cotton relies on unique fabric texture features, resulting in intense bright spots in the heatmap. In contrast, for an incorrect cotton leaf disease prediction (Figure 7), the model’s attention diffuses to leaf edges and background shadows, leading to misclassification, emphasizing the need for additional background normalization in future datasets.

The FabricSpotDefect dataset requires classifying various fabric surface defects and stains as detailed in Figure 6B. It includes five groups: Detergent Stains, Food Spots, Oil Stains, Blood Spots, and Paint Spots. The dataset features images of fabrics affected by detergent residue and paint marks. RaceNet analyzes these defects using Grad-CAM heatmaps. The analysis shows that the Detergent Stain heatmap highlights the distribution of stains across the fabric. Food residue is the main focus in the Food Spot heatmap. The model accurately identifies round stains in the Oil Stain group and detects blood residues with intense red areas in the Blood Spot heatmap. It also effectively classifies irregular paint stains using the Paint Spot heatmap.

Recent analytical findings indicate that DL procedures excel at detecting fabric defects and identifying items in textile materials. Grad-CAM applications show that the model effectively recognizes textile patterns for classification and accurately spots defects during inspection. The model’s focus on relevant regions leads to reliable predictions, making it a key tool for textile analysis. Automated fabric defect detectors enable manufacturers to improve quality control and decrease costs associated with substandard textile products. AI systems also ensure material consistency by classifying fabric composition to confirm quality standards. The use of Grad-CAM enhances user trust in DL solutions, making them suitable for industrial automation and textile quality assurance. However, in cases of incorrect predictions, such as shown in Figure 7, the attention map may become dispersed across repetitive patterns, leading to missed subtle defects and misclassifications. This emphasizes the need for explainable predictions to build confidence in automated quality control. The XCottL-FebViT provides valuable insights for practitioners during implementation, as visualizations for fabric defect detection effectively highlight areas of concern, including stains, tears, and weave disruptions. The model succeeds in accurately locating these irregularities while minimizing distractions from irrelevant background details, demonstrating strong feature localization despite the variability in textile data.

These explainability outputs help field practitioners, agronomists, and textile quality inspectors understand and verify model predictions on-site. By visualizing how the model arrives at its conclusions, users can align its focus with their expertise, which fosters trust and allows for ongoing improvements. These interpretability features also support semi-automated quality control processes, enabling human inspectors to efficiently validate automated decisions, especially in unclear cases. This enhances decision-making reliability while ensuring scalability in agricultural and industrial inspection workflows.

We assessed the effectiveness of Grad-CAM visual explanations using three saliency metrics: Pointing Game Score, Deletion AUC, and Insertion AUC. The Pointing Game Score measures how well the highest activation point matches the true disease region. Deletion AUC indicates how model confidence decreases when regions are removed, while Insertion AUC shows the increase in confidence when regions are added. As shown in Table 26, XCottL-FebViT achieves a high classification accuracy and offers reliable visual explanations, which is important for real-world applications. It scored 85.7% on the Pointing Game Score, the best alignment with disease annotations. Further, XCottL-FebViT has lower Deletion AUC and higher Insertion AUC, proving its explanations are more informative and impactful on prediction confidence compared to baselines.

**Table 26:** Quantitative evaluation of Grad-CAM explanations using Pointing Game Score, Deletion AUC, and Insertion AUC across models

Model	Pointing Game Score (%)	Deletion AUC	Insertion AUC
Nested-TNT	76.2	0.44	0.59
CrossViT	78.5	0.42	0.61
Multi-ViT	79.1	0.40	0.63
PMVT	80.3	0.39	0.65
XCottL-FebViT (Ours)	85.7	0.35	0.68

We developed a standardized data collection pipeline for detecting fabric defects using mounted cameras with controlled lighting to capture high-resolution images during production. These images are processed with the XCottL-FebViT framework for real-time quality prediction, improving defect detection, and minimizing manual inspections. We evaluated the base LEViT model, the LEViT+CNN hybrid, and the complete XCottL-FebViT model with and without the XAI module on the CottonFabricImageBD dataset under uniform conditions. The results in Table 27 show that the LEViT+CNN outperforms the base LEViT through enhanced local feature extraction for detecting texture-sensitive defects. The full XCottL-FebViT model achieves the highest performance across all metrics, confirming the effectiveness of the hybrid design. Furthermore, the XAI module provides interpretability for operators and quality control inspectors without significantly impacting classification performance, making the system suitable for industrial use.

**Table 27:** Component-wise and XAI contribution analysis for fabric quality prediction on the CottonFabricImageBD dataset

Model	F1 Score (%)	PR AUC (%)	MCC (%)	Specificity (%)
LEViT (Base)	90.5	91.2	88.7	92.0
LEViT+CNN	93.4	94.1	91.8	94.6
XCottL-FebViT (No XAI)	97.6	98.2	96.9	98.4
XCottL-FebViT (With XAI)	97.5	98.0	96.7	98.2

### Practitioner-centric evaluation of explainable AI outputs

A structured evaluation was conducted to assess the effectiveness of Grad-CAM visual explanations from XCottL-FebViT. Five domain experts (two agronomists, two textile quality engineers, and one field technician) reviewed paired original and Grad-CAM overlay images related to cotton leaf disease and fabric defects. This simulated real-world decision-making in field and factory settings, as detailed in Table 28. The experts rated the outputs on a 5-point Likert scale in three areas: (i) salient region clarity, focusing on how well highlighted areas matched disease or defect zones; (ii) confidence in prediction, assessing whether the visual explanations increased trust in the automated outputs; and (iii) workflow integration, evaluating how easily XAI outputs could fit into current processes. Qualitative feedback was also collected for insights into the integration of XAI.

**Table 28:** Structured practitioner evaluation of Grad-CAM XAI outputs

Criterion	Avg. Score	Std. Dev.	% Rated ≥ 4	Agreement (%)	Key Feedback Summary
Salient Region Clarity	4.5	0.3	100%	96%	“Heatmaps align with disease/defect regions, aiding inspection.”
Confidence in Prediction	4.3	0.5	80%	92%	“Visual focus builds trust before acting on the model’s output.”
Workflow Integration	4.0	0.6	80%	88%	“Accelerates preliminary screening while retaining manual oversight.”

The evaluation showed that practitioners found the Grad-CAM overlays effectively identified important features such as fungal lesions and defect boundaries, matching manual inspection standards. This accuracy allowed for faster verification and boosted confidence in automated classifications, making practitioners more willing to act on model predictions with clear visual explanations. The XAI outputs also streamlined operational workflows, enabling quicker preliminary screenings before in-depth manual inspections, which reduced inspection time without compromising quality. Feedback indicates that the explainability mechanisms in XCottL-FebViT enhance user decision-making, trust, accountability, and efficiency in semi-automated inspections within agriculture and textiles.

### Web application

**Figure 8:**
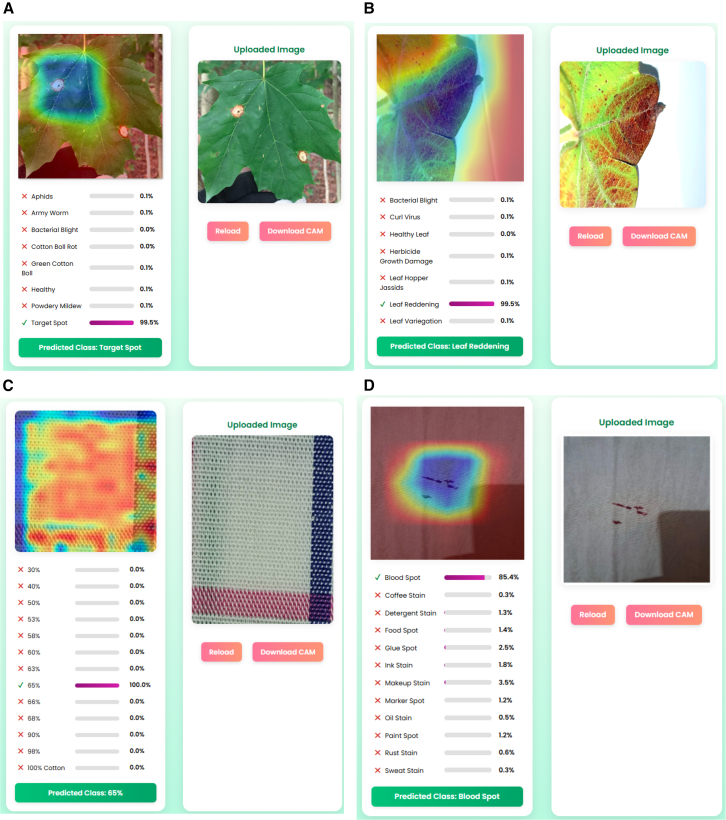
Web-based inference and explainability demonstrations The application predicts cotton leaf conditions and visualizes decision evidence using Grad-CAM: (A) Target Spot and (B) Leaf Reddening, where heatmaps localize symptomatic regions supporting the predicted class probabilities. For textile analysis, the interface similarly reports predictions and saliency maps for (C) fabric composition (e.g., 65% cotton) and (D) stain/spot defects (e.g., blood spot).

CottonVerse is a diagnostic platform that allows users to analyze cotton leaf diseases. Users can upload images of cotton leaves, which the system processes to identify potential diseases. The interface includes four sections displaying the input image, Grad-CAM visualizations, diagnostic labels with disease categories and scores, and the final prediction. For example, it identifies Target Spot disease with 100% accuracy according to Figures 8A and 8B, while also confirming Leaf Reddening as a diagnosis, showing no probability for other diseases. The model demonstrates high accuracy in classifying diseases, backed by Grad-CAM visualizations that highlight important leaf regions contributing to each classification. These heatmaps enhance model transparency and improve understanding for scientists, agronomists, and farmers.

The CottonVerse system analyzes fabric compositions with accuracy levels ranging from 30% to over 50%. The model achieves full classification trust (100%) within the 65% category for evaluated fabrics. It uses explainable Grad-CAM methods to show which image components influenced classification, aiding in decision-making. Stain identification is illustrated in Figure 8D, based on fabric images with marked stains under red light. The model tests different stains, including blood, coffee, ink, and paint. It accurately identifies stains; for example, it fully matches blood stains while dismissing other types. The model clearly indicates which areas it examined to reach its conclusions.

CottonVerse offers a user-friendly system with fabric classifiers and stain detectors, allowing users to upload and modify files after processing. Its clear interface and visual indicators make it adaptable for various needs across different business sectors. In the textile industry, the system automates quality assurance by inspecting fabric materials for defects. For cleaning and laundry services, it accurately identifies stains, helping determine the best cleaning methods. E-commerce fabric vendors can use it to validate textile materials and detect defects before shipping. The system employs deep learning-based classification and CNN for effective analysis. Future upgrades may enhance stain identification, enable potential real-time functionality, and improve classification performance across diverse fabric surfaces and lighting conditions.

### State-of-the-art comparison

The evaluation assesses various model types, including CNNs, transformer architectures (ViT, DINOv2, DAT-Net), hybrid systems, and YOLO frameworks, by analyzing dataset features and classification boundaries. Performance metrics used include accuracy scores, F1 Score, and mean Average Precision (mAP). The models are compared across multiple datasets, revealing significant differences in accuracy. GreenViT and Vallabhajosyula et al.’s model achieves 100% accuracy, while others struggle due to dataset complexities. LeViT models outperform others with F1 Scores up to 99.95%, demonstrating exceptional success in textile defect classification. Despite this, the reviewed studies in Table 29 show limited use of XAI methods. Most models lack explainability, with exceptions such as DVTXAI and an explainable version of ResNet50. LeViT-based models, integrated with XAI systems, provide better interpretation capabilities for practical applications, crucial for industrial systems requiring trustworthy decision-making. Practically, existing models struggle to operate efficiently in real-world settings. Research often prioritizes model accuracy over user-friendly requirements. Industrial classification models mostly consist of YOLO architectures paired with hybrid CNNs and transformer networks (ViT, KNN, DINOv2, HMFOCL-FDD). LeViT’s capabilities enable the deployment of automated inspection systems, with model efficiency dependent on dataset size and composition. For example, Kukadiya et al. used 402 images, while Fashion-MNIST has 60,000.

**Table 29:** A comparative analysis highlights the performance differences between existing and proposed models, demonstrating the system’s efficiency and improvements in classification accuracy

Reference	Model	Dataset	Data	Classes	Result	XAI	Application
Kukadiya et al.^12^	CNN (Custom)	Real-field dataset	402	4	96.88% accuracy	No	No
Peyal et al.^13^	Lightweight CNN	Kaggle dataset	1785	4	99.42% accuracy	Yes (Grad-CAM)	No
Rai & Pahuja et al.^14^	Improved CNN	Real-field dataset	2293	4	97.98% accuracy	No	No
Memon et al.^15^	Meta DL	Custom dataset	2385	7	98.53% accuracy	No	No
Taher et al.^16^	Custom CNN	Kaggle dataset	2637	6	99.16% accuracy	No	No
Gao et al.^24^	Transformer + KG	Custom dataset	3129	10	94% accuracy	No	Yes
Vallabhajosyula et al.^17^	Residual ViT	Three datasets	9545	51	99.7% accuracy	No	No
Remya et al.^25^	ViT + Acoustic Sensors	Custom dataset	32000	5	99% accuracy	No	Yes
Parez et al.^18^	GreenViT	Three datasets	58807	51	100%, 98%, 99% accuracy	No	No
Qiu et al.^19^	SpemNet	Three datasets	13887	–	99.03% precision	No	No
Baek^20^	Multi-ViT	Multiple leaf datasets	–	–	99.49% accuracy	No	No
Kamal et al.^21^	DVTXAI (ViT + XAI)	PlantVillage	61486	9	93.56%–99.95% accuracy	Yes (SHAP)	No
Askr et al.^22^	Explainable ResNet50	Custom dataset	2400	6	99% accuracy	Yes	No
Shao et al.^23^	CANnet (CNN)	Public + Self-Collected	3910	6	96.3%–98.6% accuracy	No	No
Niloy et al.^26^	Custom CV Model	CottonFabricImageBD	27300	13	–	No	Yes
Islam et al.^27^	YOLOv8 + COCO	FabricSpotDefect	2300	–	–	No	Yes
Hassan et al.^28^	DCNN + YOLOv8	Custom dataset	3900	13	97.49% mAP	No	Yes
Nasim et al.^29^	YOLOv8, YOLOv5, MobileNet	Chenab Textiles	2800	7	84.8% mAP (YOLOv8)	No	Yes
Kumar et al.^30^	LSTM	TILDA	–	7	96.8% accuracy	No	No
Yaşar Çıklaçandır et al.^32^	ResNet18 + KNN	Kısaoğlu & TILDA	–	6	83.1% accuracy	No	No
Meister et al.^31^	LSTM	LLSS dataset	–	–	>94% accuracy	No	No
Luo et al.^33^	YOLO-SCD	Aliyun Tianchi	5096	12	82.92% mAP	No	Yes
Wang et al.^34^	Cascade R-CNN + AFAM	CloF + MS-COCO	5913	20	+2.6 AP50	No	Yes
Alruwais et al.^35^	HMFODL-FDD (Hybrid CNN)	Custom dataset	415	3	95.47% accuracy	No	No
Smith et al.^36^	ViT	Leather Defect classification	–	–	High accuracy	No	No
Shang et al.^37^	DAT-Net (Transformer)	Three datasets	4060	–	86.86%–90.19% mIoU	No	No
Liu et al.^38^	M-VIT-DDQ	Infrared Thermography	–	–	Higher accuracy than CNN/RNN	No	No
Wang et al.^39^	Defect Transformer (DefT)	Three datasets	6436	–	98.08% accuracy	No	No
Abd Alaziz et al.^40^	ViT + DINOv2	Fashion-MNIST + Kaggle	60000	10	95.25%–98.53% accuracy	No	Yes
Zhou et al.^41^	ETDNet (Transformer)	Three datasets	15276	–	46.7% AP, 80.2% AP50	No	Yes
Ours	LEViT	CottonLeafNet	8,168	8	99.80 ± 0.62 F1 Score	Yes (Grad-CAM)	Yes
Ours	LEViT	SAR-CLD-2024	7,000	7	99.60 ± 0.52 F1 Score	Yes (Grad-CAM)	Yes
Ours	LEViT	CottnFabricImageBD	13,000	13	99.72 ± 0.45% F1 Score	Yes (Grad-CAM)	Yes
Ours	LEViT	FabricSpotDefect	6,000	12	99.95 ± 0.40% F1 Score	Yes (Grad-CAM)	Yes

Our study demonstrates versatility using datasets such as CottonLeafNet and SAR-CLD-2024 with CottonFabricImageBD and FabricSpotDefect. LeViT models achieved the highest performance with a 99.95% F1 Score. Although XAI adoption is low, it provides high accuracy while lacking essential explainability. Most industrial applications use YOLO and hybrid CNN models or ViT architectures, yet many models remain in academic research. Larger datasets facilitate the creation of robust, generalized models.

## Discussion

The LEViT model delivers advanced classification precision, improved computational speed, and dependable operation for cotton leaf disease and fabric defect classification. LEViT delivers superior performance to other CNN-based systems and ViT-based architectural models. The particular strength of LEViT lies in its ability to perform exceptionally well on CottonLeafNet images while maintaining reduced processing time suitable for IoT applications in industrial automation. Performance gains arise mainly from data augmentation methods that the system employs for extensive use. The implemented strategies succeeded in reducing overfitting problems with insufficient data and domain adaptation limitations. Multiple implementation difficulties persist with the LEViT framework, including its deep computational nature and its sensitivity to domain variations. They also need extensive datasets, difficult interpretation, and an inadequate combination of different data types. These areas require further investigation.

Data augmentation supported better generalization ability and environmental condition robustness in LEViT operations. The training set augmentation enabled LEViT to identify invariant and discriminative features because DL models typically need large volumes of data for proper operation. Part of the research employed geometric transformations for augmentation, including random rotations and translations, and flipping and affine transformations to enhance model performance in various perspectives. Besides gamma corrections, light adjustment techniques that modify brightness and contrast helped the LEViT model excel with standard variation in various atmospheric conditions. Implementing elastic distortions combined with cutout modifications involving grid warping and random masking elements helped make the model more tolerant to natural imaging imperfections and partial image losses. The last approach used GAN-based synthetic augmentation to address data imbalance concerns regarding rare disease manifestations and unobtrusive textile faults. Through this approach, the model adequately learned proper representations of minority classes. Each augmentation method strengthened LEViT’s capability for dealing with unforeseen test distributions, thus reducing overfitting issues and improving performance.

Traditional disease recognition and defect finding through DL mainly use CNNs, but these architectures do not perform well when dealing with a worldwide contextual understanding of datasets. The self-attention mechanisms in ViTs solve the lack of long-distance relationship classification by enabling model analysis across the entire image field. The normal operation of ViTs results in computational complexity that grows with an O(n^2^) rate, thus becoming inappropriate for potential real-time tasks on devices with limited resources. The LEViT model adopts hierarchical CNN features and Transformer self-attention to combine nearby efficiently and complete visual understanding. LEViT adopts a hierarchical self-attention approach for dynamic spatial and channel attention integration across tokens. In contrast, Nested-TNT uses hierarchical tokenization yet remains calculating-intensive, and CrossViT runs independent multi-scale patch operations. The model delivers strong feature results through efficient computation management.

LEViT demonstrates suitability for industrial automation and precision agriculture applications because its enhanced generalization and efficiency meet specific requirements. Accurate identification of cotton leaf diseases depends heavily on detecting delicate signs of pathology through different environmental situations to enable prompt response methods. Adopting XAI through Grad-CAM visualizations enables agronomists and farmers to check AI-generated predictions, which helps establish confidence and openness regarding AI-enabled disease identification systems. LEViT proves successful in fabric defect classification since it detects micro-defects within high-resolution textile images while reaching high accuracy rates and avoiding incorrect classifications. The lightweight design structure of LEViT supports IoT deployments for potential real-world defect monitoring in smart manufacturing; therefore, it reduces manual inspection work while improving quality control effectiveness.

To deploy XCottL-FebViT on lower-end hardware with limited resources, several model compression techniques can be utilized. Quantization, such as post-training INT8 or FP16, can reduce model size and speed up inference without sacrificing accuracy. Pruning, both structured and unstructured, can eliminate unnecessary weights and channels, lowering memory use and computational demands. Knowledge distillation can create a smaller student model that mimics XCottL-FebViT’s performance, making it suitable for resource-limited settings. Additionally, using lightweight frameworks such as TensorRT, TFLite, and ONNX Runtime will enable efficient, low-power inference on embedded and edge devices, aiding deployment for agricultural and industrial inspections.

Recent research has focused on creating compact ViT architectures that are low in parameters and FLOPs, suitable for edge-device applications without sacrificing accuracy. MobileViT combines MobileNetV2 with small transformer modules, achieving under 3 million parameters and low FLOPs while maintaining competitive accuracy. EfficientFormer employs a pure transformer structure with a MetaBlock to minimize memory and computation, showing solid performance for mobile environments. EdgeViTs enhance efficiency by integrating local depth-wise convolutions with sparse self-attention blocks. ViT-Lite and TinyViT reduce computational demands by minimizing attention heads and simplifying transformer blocks. The DeiT model employs knowledge distillation to create lightweight ViT versions without performance loss. XCottL-FebViT connects lightweight ViTs to practical applications, effectively detecting cotton diseases and classifying fabric defects on resource-limited devices. While MobileViT and EdgeViTs excel in speed and low computational needs, their parameter reduction may affect sensitivity to subtle textures important for identifying cotton leaf diseases. EfficientFormer may need further tuning for specific tasks. In contrast, XCottL-FebViT uses the LEViT backbone, merging CNN-based feature extraction with transformer self-attention to maintain global context while reducing costs. It includes a lightweight Grad-CAM-based explainable AI head for interpretability, essential for agriculture and textile quality control. The model effectively preserves local texture extraction through convolutional blocks while leveraging transformers for global reasoning, making it robust against variations that could impact other models.

### Limitations of the study

XCottL-FebViT effectively classifies cotton leaf diseases and fabric defects, but faces challenges due to existing datasets and system constraints. Datasets such as CottonLeafNet and SAR-CLD-2024 mainly consist of samples from South Asia, which can lead to domain shift when models are used in different regions with varying cultivars, soil types, or climates. Furthermore, these datasets often lack real-world variations such as motion blur, occlusions, shadows, and inconsistent lighting, which are common in agricultural and industrial settings. With sizes ranging from 2,000 to 10,000 images, they are insufficient for training large transformer models and suffer from class imbalance for rare disease types, hindering generalization. Future efforts should focus on collecting geographically diverse data that captures different environmental conditions and lighting. Expanding the dataset to include underrepresented classes and utilizing better sampling techniques or synthetic data generation will enhance model robustness and fairness.

While XCottL-FebViT eases real-world deployment, further engineering and validation are required for practical use in agriculture and industry. In manufacturing, latency and throughput are crucial, particularly for high-speed inspection systems needing over 30 FPS at high resolutions. Although the model shows real-time inference in controlled tests, hardware optimizations such as TensorRT, quantization, or pruning may be necessary for production-level performance. Low-power ARM-based edge devices present additional constraints that require further optimization. Additionally, integrating with existing inspection systems poses challenges, including compatibility with camera hardware, lighting, and operator interfaces. To build operator trust and improve usability, incorporating Grad-CAM–based explanations into user-friendly dashboards will necessitate collaborative design.

To ensure practical readiness, future work will focus on collaborations with the textile and agricultural industries for real-world testing. In textiles, pilot factory trials will assess defect detection under realistic conditions. In agriculture, partnerships with farms and extension services will facilitate testing with mobile devices in field environments. These efforts will gather more representative datasets, evaluate system usability, and enhance visualization interfaces based on practitioner feedback, ultimately guiding further improvements for effective deployment. LEViT, such as other ViTs, has a scaling pattern in self-attention that makes processing large-scale or high-resolution data computationally intensive. The multi-head self-attention and deep MLP layers lead to high resource use, complicating deployment on limited hardware. Efficient transformer variants such as Linformer, Nyströmformer, and Performer, can address these challenges through low-rank self-attention approximations. Further, techniques such as structured pruning, quantization-aware training, and knowledge distillation can reduce model complexity and latency. Despite improved domain generalization, LEViT is sensitive to variations in lighting, occlusions, and image distortions. Future work should implement adversarial robustness strategies such as projected gradient descent (PGD), fast gradient sign method (FGSM), and TRADES-based training, to enhance stability under disruptions.

LEViT requires large labeled datasets due to its lack of CNN-style inductive biases, making it less effective in low-data situations. Integrating self-supervised learning (SSL) techniques, such as contrastive learning frameworks (SimCLR, MoCo, MAE), will improve feature extraction from unlabeled data, reducing reliance on manual labeling. Moreover, few-shot learning methods such as model-agnostic meta-learning (MAML), prototypical networks, and relation networks could enhance the recognition of rare disease and defect classes. A limitation of ViTs is their low interpretability. Although saliency-based methods such as Grad-CAM, SHAP, and LIME, help with visualization, they often miss fine-grained details in high-resolution images. Future research should focus on concept-level interpretability frameworks and attention-visualization tools to better understand the relationship between self-attention weights and feature importance. Finally, LEViT only processes RGB images, limiting its multimodal inspection capabilities. Incorporating additional sensory modalities, such as hyperspectral, infrared, or acoustic data, could improve detection reliability. Multimodal fusion frameworks such as M3Fusion, CLIP, or ALIGN, paired with cross-modal contrastive learning, may enhance multisensor robustness and interpretability. These developments will steer XCottL-FebViT toward a more efficient, interpretable, and deployment-ready solution for agricultural and industrial visual inspection.

## Resource availability

### Lead contact

Information and requests for resources should be directed to and will be fulfilled by the lead contact, Abhishek Appaji (am.appaji@maastrichtuniversity.nl).

### Materials availability

The study did not generate new unique reagents.

### Data and code availability

Data: The datasets used in this study are publicly available and sourced from CottonLeafNet,^42^ SAR-CLD-2024,^43^ CottonFabricImageBD,^44^ and FabricSpotDefect.^45^

Code: Source code of the study is available at https://github.com/rezaul-h/CottonVerse.

Additional information: Any additional information required to reanalyze the data reported in this article is available from the lead contact upon request.

## Acknowledgments

This work received no specific funding and was supported by institutional computing resources. The authors thank the dataset contributors of CottonLeafNet, SAR-CLD-2024, CottonFabricImageBD, and FabricSpotDefect for providing open-access resources that enabled this research. We also appreciate the valuable feedback from reviewers and colleagues, which helped refine the article.

This research did not receive any specific grant from funding agencies in the public, commercial, or not-for-profit sectors.

## Author contributions

Experimental design and conceptualization: S.M.M.R.S., R.H., and A.A. Data collection and curation: A.S.U.K.P. and A.H. Software development, model implementation, and formal analysis: A.S., A.A.S., M.R.A., and A.A.N. Visualization, validation, and investigation: J.D., A.A.S., A.H., and R.H. Writing – original draft preparation: S.M.M.R.S., A.S., A.S.U.K.P., and J.D. Writing – review and editing: all authors. Supervision, resource provision, and funding acquisition: A.A. All authors have read and approved the final article.

## Declaration of interests

The authors declare no competing interests.

## STAR★Methods

### Key resources table

**Table:** 

REAGENT or RESOURCE	SOURCE	IDENTIFIER
**Deposited data**		

CottonLeafNet dataset	Public repository	URL: https://www.kaggle.com/datasets/rezaullhaque/cottonleafnet-dataset
SAR-CLD-2024	Public repository	URL: https://www.kaggle.com/datasets/sabuktagin/dataset-for-cotton-leaf-disease-detection
CottonFabricImageBD	Public repository	URL: https://data.mendeley.com/datasets/3vc56ddjhw/2
FabricSpotDefect	Public repository	URL: https://data.mendeley.com/datasets/6574nhzm8x/1
Code and web application	This study	URL: https://doi.org/10.5281/zenodo.18476909

**Software and algorithms**		

XCottL-FebViT	This study	Hyperparameters and training details reported in Table 10
Python version 3.10.12	Python Software Foundation (PyPI)	https://www.python.org/downloads/release/python-31012/
PyTorch version 2.2.2	PyPI	https://pytorch.org/get-started/previous-versions/
TorchVision version 0.17.2	PyPI	https://pypi.org/project/torchvision/
Timm version 0.9.16	PyPI	https://pypi.org/project/timm/0.9.16/
NumPy version 1.26.4	PyPI	https://pypi.org/project/numpy/1.26.4/
Pandas version 2.2.2	PyPI	https://pypi.org/project/pandas/2.2.2/
scikit-learn version 1.4.2	PyPI	https://pypi.org/project/scikit-learn/1.4.2/
Matplotlib version 3.8.4	PyPI	https://pypi.org/project/matplotlib/3.8.4/
OpenCV version 4.9.0.80	PyPI	https://pypi.org/project/opencv-python/
Albumentations version 1.4.3	PyPI	https://pypi.org/project/albumentations/
pytorch-grad-cam version 1.5.2	PyPI	https://pypi.org/project/pytorch-gradcam/
ONNX version 1.16.0	PyPI	https://pypi.org/project/onnx/
ONNX Runtime GPU version 1.17.1	PyPI	https://pypi.org/project/onnxruntime-gpu/

**Other**		

GPU hardware for training/evaluation	NVIDIA RTX A6000	Reported in Table 17
Inference/efficiency benchmarks	This study	See Tables 18, 19, and 20

### Experimental model and study participant details

This study solely focused on image analyses of cotton plant leaves and textile fabrics and did not involve human participants, identifiable personal data, vertebrate animals, or live experimental organisms. Therefore, no institutional review or animal ethics approval was needed.

### Method details

#### Cotton leaf disease identification datasets and preprocessing

We evaluated experimental models for classifying leaf diseases using two publicly available datasets. The first dataset, CottonLeafNet,^42^ contains 4,979 images depicting various conditions of leaf diseases and healthy leaves, covering multiple disease types. The images were captured using a Sony DSLR, an Apple iPhone 8, and a Vivo Y21 smartphone, resulting in variations in quality and lighting. Each image is meticulously labeled to ensure accurate categorization. The dataset includes front and back views of leaves, providing detailed visual information about symptoms. It consists of eight disease classes with a balanced distribution: 836 images of aphids, 837 of army worms, 840 of bacterial blight, 1,021 of cotton boll rot, 939 of green cotton bolls, 839 of healthy leaves, 838 of powdery mildew, 829 of target spots. A summary of the class distribution can be found in Table 1. The images were collected under varying environmental conditions, including daytime and nighttime, sunny weather, and rain, contributing to exposure differences. Figure 2A displays sample images from each category.

Preprocessing was applied to improve disease visibility and robustness to variations in real-world data collection. To standardize pixel values, min-max normalization was applied, computed as Equation 1. Where X represents the original pixel intensity, and $X_{\min},X_{\max}$ denote the minimum and maximum intensity values, respectively.

(Equation 1) $$X_{\text{norm}}=\frac{X-X_{\min}}{X_{\max}-X_{\min}}$$

As shown in Table 3, a random rotation of ±25° was applied to account for variations in leaf orientation. Brightness adjustment (range: 0.8 to 1.2) was introduced to mimic real-world lighting conditions, while Gaussian blur (kernel size: 3 × 3) simulated minor focus imperfections. Furthermore, elastic deformation (Alpha = 34, Sigma = 5) was implemented to mimic the natural bending of leaves. CottonLeafNet data distribution appears in Table 2. A CottonLeafNet dataset sample image demonstrates its various variations resulting from rotation modifications, brightness alterations, Gaussian blurring, horizontal and vertical transformations, and elastic intensity changes, as illustrated in Figure 3.

National Cotton Research Institute in Gazipur, Dhaka, Bangladesh conducted experiments from October 2023 to January 2024 to create the SAR-CLD-2024 dataset.^43^ Supervised by agricultural experts, the team collected high-quality photographs of cotton diseases under different lighting conditions and weather circumstances. The dataset includes 2,137 images across seven categories, featuring both diseased and healthy cotton leaves. Specifically, it contains 250 images of Bacterial Blight, 431 of Curl Virus, 280 showing Herbicide Growth Damage, 225 of Leaf Hopper Jassids, 578 of Leaf Reddening, 116 of Leaf Variegation, and 257 of Healthy Leaves. A total of 1409 original disease images comprise the SAR-CLD-2024 database according to Table 4, highlighting symptoms such as discoloration, necrosis, and damage. Photos were captured using a Redmi Note 11S smartphone with resolutions of 3000 × 4000 pixels, 2239 × 2239 pixels, and 1597 × 1597 pixels, providing critical details for disease identification, as illustrated in Figure 2B.

Histogram equalization is used to improve contrast as Equation 2, where $H_{\text{eq}}\left(i\right)$ represents the equalized histogram intensity, h(j) is the original histogram, and N is the total number of pixels. Random shear angles of ±15° served as leaf distortion approximation methods according to Table 3. The close-up views of diseased leaves were simulated by applying specific transformations at rates between 0.9 and 1.1. A combination of gamma factors ranging from 1.2 to 1.5 was introduced to enhance the visibility of disease indicators. The distribution of observations after augmentation exists in Table 5. The figure within the SAR-CLD-2024 dataset displays modified perspectives that result from combining random shearing with zoom-in transformation, contrast enhancement, and rotation followed by horizontal flipping as demonstrated in Figure 5.

(Equation 2) $$H_{\text{eq}}\left(i\right)=\frac{\sum_{j=0}^{i}h\left(j\right)}{N}$$

#### Fabric defect identification datasets and preprocessing

The CottonFabricImageBD dataset^44^ collected fabric samples from major textile markets in Dhaka, including Bashundhara Shopping Complex, Bongo Bazar, Islampur, and Mouchak. High-resolution images were captured using Samsung M12 smartphones with 48 MP cameras under standardized lighting and fixed imaging distances to ensure uniformity and minimize shadows. A textile engineer verified dataset accuracy by testing cotton content with thread-counting machines. The dataset comprises 1,300 images across 13 cotton-percentage categories (each with 100 images) ranging from 30% to 99%, ensuring balanced and diverse representation. Each 900 × 1200 RGB JPG image provides detailed fabric texture. Thread density was evaluated horizontally and vertically against 100% cotton standards, with cotton content calculated using Equation 3. Table 6 lists the image frequency for each cotton-percentage range in the classification system.

(Equation 3) $$\text{Cotton}\;\text{Percentage}=\left(\frac{\text{Thread}\;\text{Count}\;\text{of}\;\text{Sample}\;\text{Fabric}}{\text{Thread}\;\text{Count}\;\text{of}\;\text{Ideal}\;\text{Cotton}\;\text{Fabric}}\right)\times 100$$

The accuracy of cotton composition evaluation depends on fabric textures. The process of converting images to grayscale or LAB color space performed two tasks by improving pattern details yet minimizing extraneous background clutter. Figure 2C depicts the sample image from each class of the dataset. Feature extraction is performed using the Gray-Level Co-occurrence Matrix (GLCM) as Equation 4, where P(i,j) represents the probability of intensity values i and j occurring at a distance d in direction θ. As shown in Table 3, CLAHE (Clip Limit = 2.0, Grid size = 8 × 8) was applied to enhance contrast. The scanner noise simulation method added Gaussian noise with a variance of 0.01 after doing fine detail texture preservation through sharpness adjustment (Factor = 1.5). After augmentation, the data distribution of CottonFabricImageBD appears in Table 7. A sample CottonFabricImageBD image appears with augmented effects in Figure 7 that demonstrate contrast enhancement with Gaussian noise addition, sharpness adjustment, affine transformation, and color jittering.

(Equation 4) P(i,j|d,θ)

The FabricSpotDefect dataset^45^ comprises 1,014 expertly labeled high-resolution fabric images captured under controlled household lighting to simulate inspection conditions in retail and domestic environments. Data were collected using three Samsung smartphone models: Galaxy Note20, S20 FE, and A53 5G to enhance variability and robustness across camera profiles. As shown in Figure 2D, it includes diverse fabric types such as cotton, linen, silk, denim, jacquard, and patterned textiles for comprehensive defect classification. The Spot Defects category contains all identified defects, classified into twelve distinct labeled types, with image counts for each category listed in Table 8.

The annotation of each defect category happened by textile experts who conducted it manually to achieve high precision and uniformity in labeling. The database contains JPG images, which maintain a 416 × 416 pixel resolution format to support DL-based defect classification software. The dataset splits into three sections where 643 images function for training purposes, 219 images exist for validation, and 152 images serve testing needs. Through its ability to process various textile fabrics, the DL models gain comprehensive learning capabilities that aid defect pattern classification on multiple textile materials. The Roboflow tool used manual labeling to identify spot defects through bounding boxes and polygon segments.

For the FabricSpotDefect dataset, preprocessing aimed to enhance defect visibility and improve robustness under various manufacturing and inspection conditions. Min-max normalization standardized pixel intensities, while adaptive thresholding (Equation 5) improved defect segmentation, where T(x,y) denotes the threshold at pixel (x,y), μ(x,y) is the local mean intensity, and C is a constant for binarization tuning. Data augmentation included random shadow distribution and brightness adjustments (0.7–1.3) to simulate uneven factory lighting (Table 3). Elastic transformation (Alpha = 40, Sigma = 6) introduced realistic stretch effects while maintaining defect classification accuracy. Besides, salt-and-pepper noise (probability = 0.02) simulated sensor imperfections. The augmented data distribution is shown in Table 9.

(Equation 5) T(x,y)=μ(x,y)+C

#### Nested-TNT model execution

It is a hierarchical deep learning model designed to extract both local and global visual patterns through a multi-level tokenization process. It consists of inner and outer transformer operations. The inner block focuses on fine-grained image patches, which are enhanced through linear projections and positional embeddings. These patches are then processed with self-attention mechanisms to capture localized features, such as disease lesions or fabric imperfections. The outer transformer block aggregates these refined tokens into higher-level representations, akin to sentences, enabling the model to capture broader spatial dependencies across the image. This two-stage design enables the fusion of micro- and macro-level visual cues, thereby enhancing performance on complex classification tasks. As illustrated in Figure 3A, the architecture’s structured token hierarchy effectively recognizes subtle plant disease symptoms and minor fabric defects. While Nested-TNT demonstrates strong generalization capabilities through its hierarchical attention mechanism, its layered complexity and computational demands limit its scalability for real-time or resource-constrained applications. These challenges highlight the need for more lightweight and efficient alternatives.

#### CrossViT model execution

Vision Transformers (ViTs) excel at capturing long-range dependencies in image patches, but their fixed patch size limits feature extraction at different spatial scales.^46^ CrossViT overcomes this by using a dual-branch architecture: a Small Patch Size Branch (S Branch) for detailed feature extraction and a Large Patch Size Branch (L Branch) for broader structural understanding.^47^ As visualized in Figure 3B, Each branch applies a linear projection to its image patches and uses an independent Transformer encoder. A cross-attention mechanism merges the information, enabling the model to learn complementary features. The final predictions are generated through Multi-Layer Perceptron (MLP) heads. This architecture captures both localized patterns and global structures effectively, making CrossViT suitable for tasks like cotton leaf disease classification and fabric quality analysis. However, its dual-branch processing and multi-head attention result in substantial computational overhead, limiting its application in edge or real-time settings. Our proposed XCottL-FebViT builds upon the concept of multiscale attention but integrates it into a more compact, single-branch framework. By leveraging CNN-based hierarchical features and a lightweight Transformer backbone (LEViT), our model preserves multiscale sensitivity while drastically improving efficiency and scalability.

#### Multi-ViT model execution

This architecture builds upon traditional ViT models by incorporating parallel Transformer branches, with each branch processing image patches independently to extract diverse feature representations. This parallelism enhances the model’s ability to generalize across various datasets, such as those related to cotton leaf diseases, fabric defects, and fabric composition, by capturing a richer set of spatial and contextual cues. In the initial phase, input images are divided into smaller patches, which are then subjected to high-dimensional projection and positional embedding. Each ViT branch performs multi-head self-attention, followed by normalization and MLP layers for feature transformation. The independently processed features are subsequently aggregated using normalized attention weighting, resulting in a unified representation for final classification. Figure 3C illustrates the complete Multi-ViT pipeline. This architecture supports redundancy-tolerant decision-making, allowing each branch to operate independently while collaboratively contributing to more robust and interpretable outputs.^48^ Its weighted feature aggregation strategy improves classification precision and reduces the risk of overfitting through diversified attention. However, the computational demand of multiple parallel ViT branches, each requiring separate attention and feed-forward layers, presents efficiency challenges, particularly in resource-constrained environments. To address these limitations, our proposed model combines the advantages of multi-branch attention with a more compact and unified design.

#### PMVT model execution

The Patch-Merged Vision Transformer (PMVT) is a hybrid deep learning architecture that integrates CNN-based local feature extraction with ViT encoding. This combination is designed to effectively classify cotton leaf diseases, estimate fabric composition, and detect imperfections in textiles.^49^ The model utilizes Convolutional Block Attention Modules (CBAM) to focus on important spatial and channel-specific features before passing the processed feature maps to the transformer encoder.^50^ Initially, input images undergo processing through stacked Conv2D layers (1 × 1 and 3× 3) to extract spatial features while maintaining structural integrity. These feature maps are then converted into tokens and enhanced using CBAM, which reduces the impact of irrelevant regions while emphasizing critical patterns. The refined tokens are sent to the Vision Transformer Encoder (VTE), which includes positional encodings, multi-head self-attention (MHA), layer normalization, and MLP blocks. This structure allows the model to learn both local textures and global dependencies, thereby enhancing classification performance across fine-grained visual domains.^51^

The final classification layers employ residual-enhanced Conv2D operations, supported by MaxPooling and AveragePooling. This approach amplifies the significance of key features and promotes a smoother gradient flow. As illustrated in Figure 3D, PMVT effectively merges CNN-driven locality with Transformer-based contextual reasoning, enabling high-precision classification for both agricultural and industrial tasks.^52^ Despite its strong interpretability, PMVT’s multi-stage architecture and attention-heavy modules lead to increased inference time and hardware requirements, which can limit deployment on edge devices or in situations where low latency is critical. In response, our XCottL-FebViT model retains the hybrid design philosophy of PMVT but significantly reduces complexity by integrating lightweight LEViT transformers with CNN-based hierarchical extraction.

#### Proposed XCottL-FebViT framework execution

In this proposed XCottL-FebViT model, LEViT model combines self-attention methods and CNNs to enhance its functionality.^53^ It operates locally while balancing execution speed and accuracy, making it well-suited for classifying cotton leaves and fabrics. Precise pattern identification is essential for detecting defects and estimating percentages. As shown in Algorithm S1, LEViT processes in a specific order: it first uses CNN features, followed by multiple transformer stages with attention components.^54^ The convolutional layers input images sized 3 × 224 × 224, extracting low-level features through three consecutive 3 × 3 convolutional layers at each of four steps. This preprocessing generates strong spatial information for the transformer blocks. The hierarchical self-attention module takes input from the CNN block output (256 × 14 × 14) and processes it through an MLP layer and 4-headed self-attention mechanisms. The architecture employs complex attention layers with six, eight, and twelve heads to enhance feature representation. The Shrink Attention Mechanism reduces dimensions while preserving important spatial information, producing a compatible feature output of 512 x 4 x 4. This attention-based approach allows the model to recognize connectivity patterns and details vital for assessing cotton leaf health and fabric quality.

The average pooling operation converts feature maps into a 512-dimensional vector, which a supervised classifier uses to determine the probability distributions for different output classes related to cotton leaf diseases, fabric flaws, and material percentages. This system includes multiple output classification structures to handle various labeling needs in commercial applications. The LEViT architecture combines CNN elements (shown in Figure 4 and starts with convolutional layers to extract spatial features, followed by hierarchical self-attention layers and MLP components for feature enhancement. LEViT excels in analyzing cotton leaf percentages and identifying fabric defects. It merges convolutional and transformer layers for fast processing, making it suitable for real-time applications in agriculture and textiles. The system captures complex patterns, intricate textures, and imperfections using strong extractive features. LEViT outperforms traditional ViTs in compactness and efficiency, achieving similar accuracy with fewer parameters, leading to faster training and inference times.^55^ It provides reliable classification outputs across various lighting conditions, fabric orientations, and surface textures by effectively generalizing information.

#### Hyperparameter tuning optimization for proposed method

In this study, we conducted a systematic hyperparameter search, detailed in Table 10. For the learning rate, we tested {1e-6, 5e-6, 1e-5, 5e-5, 1e-4} and found that 1e-4 offered the best convergence speed and stability. Higher values caused instability, while lower rates slowed learning. For batch sizes, we selected 128 from {64, 128, 256}, as it balanced gradient stability and memory efficiency. In regularization, we found that a dropout rate of 0.5 effectively prevented overfitting given our model’s complexity. Of the optimizers we compared Adam, AdamW, and LAMB and AdamW performed best due to its decoupled weight decay, enhancing generalization. We tested weight decay values {1e-6, 1e-5, 5e-5, 1e-4} and found that 5e-5 offered optimal generalization without overly restricting learning dynamics. For the learning rate schedule, Cosine Annealing provided smoother convergence and better validation performance. Adding 1000 warm-up steps helped stabilize early training.

To determine optimal training duration, we experimented with max epochs {50, 100, 200} and settled on 100, as 50 caused underfitting and 200 yielded minimal improvements. We applied early stopping with a patience value of 10 to prevent unnecessary training. Gradient clipping of 1.0 was effective in preventing gradient explosion. For architectural hyperparameters, we tested patch sizes {8 × 8, 16× 16, 32× 32} and found 16× 16 optimal for spatial detail and efficiency. Head sizes of 64 best maintained expressiveness without excess parameters. In our Transformer setup, 12 layers outperformed 6 (too shallow) and 24 (too complex). Eight attention heads struck the right balance between expressivity and computational overhead. Lastly, Xavier initialization yielded the most stable training behavior among the strategies we evaluated.

### Quantification and statistical analysis

The proposed model’s performance evaluation utilized a complete set of metrics to examine its effectiveness. The Micro F1-score (Equation 6) aggregates performance across all classes by summing class-wise true positives, false positives, and false negatives. This harmonic mean formulation ensures balanced consideration, especially in the presence of class imbalance, thereby validating the model’s sensitivity to minority class performance.

(Equation 6) $$\text{Micro}\;F1=\frac{\sum_{i=1}^{n}\left(2\cdot TP_{i}\right)}{\sum_{i=1}^{n}\left(2\cdot TP_{i}+FP_{i}+FN_{i}\right)}$$

Here, $TP_{i}$, $FP_{i}$, and $FN_{i}$ denote the true positives, false positives, and false negatives for class i, respectively, and n is the total number of classes. The Precision-Recall Area Under the Curve (PR AUC) generates quantitative assessments of model performance by computation-specific areas under Precision-Recall curves for more precise evaluation of classification quality, especially with imbalanced data. The MCC (Equation 7) provides a balanced measure even when the classes are of different sizes. It incorporates all four confusion matrix components for binary classification. For multiclass scenarios, we compute class-wise binary confusion matrices in a one-vs-rest manner and average the MCC across all classes. Where, TP, FP, TN, and FN refer to the respective counts for each class in the one-vs-rest setup.

(Equation 7) $$\text{MCC}=\frac{\left(TP\cdot TN\right)-\left(FP\cdot FN\right)}{\sqrt{\left(TP+FP\right)\left(TP+FN\right)\left(TN+FP\right)\left(TN+FN\right)}}$$

The Specificity metric quantifies the model’s ability to correctly identify true negative instances. It is also calculated using an one-vs-rest strategy for each class i, where the current class is treated as the positive class and all others as negative. The specificity for class i is shown in Equation 8. The macro-average specificity is then computed as the unweighted mean over all classes using Equation 9.

(Equation 8) $${\text{Specificity}}_{i}=\frac{TN_{i}}{TN_{i}+FP_{i}}$$

(Equation 9) $$\text{Macro}\;\text{Specificity}=\frac{1}{n}\sum_{i=1}^{n}{\text{Specificity}}_{i}$$

We used a stratified K-fold cross-validation with K=10 to assess model performance on imbalanced multiclass datasets. This approach preserves the original class distribution in each fold, which is crucial for our data. The dataset was split into 10 subsets, each representing each class proportionally. In each iteration, one fold was used for validation while the remaining 9 folds were for training. This process was repeated 10 times, ensuring each fold was validated once. The final metrics were the average of all folds.

For between-model comparisons, we conducted two-tailed paired t-tests on the per-fold scores (MCC and F1). Improvements were considered statistically significant at a p-value of less than 0.05. As summarized in Table 15, XCottL-FebViT achieved significant gains; for instance, the comparison of SAR-CLD-2024 against PMVT yielded p-values of 0.007 (MCC) and 0.010 (F1), while other baseline models showed p-values ranging from 0.011 to 0.038. To assess robustness under field and factory perturbations, we applied several conditions: (i) brightness shifts of ± 25%, (ii) Gaussian noise with a standard deviation of σ =0.05, and (iii) a combination of both factors. F1 was the primary metric for summarizing results. XCottL-FebViT experienced only a 4.1% to 5.9% drop in F1 score, whereas transformer baselines demonstrated a degradation of 8% to 10% under the same conditions (as shown in Table 16).

We quantified the effect of data augmentation through an ablation study. We began with the full pipeline and systematically removed each augmentation to measure changes in Specificity, F1, and MCC across all four datasets. Removing elastic deformations or random rotations resulted in a reduction of F1 by 1.3% to 1.8%, highlighting their contribution to invariance and generalization. Overall, the complete augmentation pipeline yielded the best performance, as detailed in Tables 21 and 22.

## Supplemental information

Document S1. Figures S1–S5 and Algorithm S1

mmc 2: All suppliment Materials.pdf
