# Supplementary material for: Explainable transformer framework for fast cotton leaf diagnostics and fabric defect detection
Source: iScience. 2025 Dec 11;29(2):114411. doi: 10.1016/j.isci.2025.114411 (PMC12915276; doi:10.1016/j.isci.2025.114411)
Supplement: Document S1. Figures S1–S5 and Algorithm S1 [file mmc1.pdf]

iScience, Volume *29*

Supplemental information

Explainable transformer framework for fast cotton leaf diagnostics and fabric defect detection

S M Masfequier Rahman Swapno, Anamul Sakib, Al Shahriar Uddin Khondakar Pranta, Amira Hossain, Jesika Debnath, Abdullah Al Noman, Abdullah Al Sakib, Md. Redwan Ahmed, Rezaul Haque, and Abhishek Appaji

S1:

Algorithm 1: XCottL-FebViT for Cotton Leaf and Fabric Defect Classification.

Input: Input image $I \in \mathbb{R}^{H \times W \times C}$

Output: Predicted class label $\hat{y}$ and GradCAM explanation heatmap H

// Step 1: Preprocessing

$I_{\text{norm}} \leftarrow \text{Normalize}(I)$; // Min-max normalization

$I_{\text{aug}} \leftarrow \text{ApplyAugmentation}(I_{\text{norm}})$; // Dataset-specific augmentations

// Step 2: Hierarchical CNN-based Feature Extraction

$F_0 \leftarrow \text{Conv3x3}(I_{\text{aug}})$; // Initial low-level features

for $l = 1$ **to** L_{cnn} **do**

  $F_l \leftarrow \text{ConvBlock}(F_{l-1})$; // Stacked CNN layers to extract spatial features

$F_{\text{cnn}} \leftarrow F_{L_{\text{cnn}}}$

// Step 3: LEViT Transformer Encoding

$T_0 \leftarrow \text{Tokenize}(F_{\text{cnn}})$; // Patch-wise token embedding

for $l = 1$ **to** L_{levit} **do**

  $T_l \leftarrow \text{MHSA}(T_{l-1})$; // Multi-Head Self-Attention

  $T_l \leftarrow \text{MLP}(T_l)$; // Feed-forward projection

  if *ShrinkLayer(l)* **then**

    $T_l \leftarrow \text{Shrink}(T_l)$; // Spatial downsampling to reduce complexity

$F_{\text{levit}} \leftarrow T_{L_{\text{levit}}}$

// Step 4: Classification

$F_{\text{global}} \leftarrow \text{GlobalAvgPool}(F_{\text{levit}})$; // Reduce to vector representation

$\hat{y} \leftarrow \text{Softmax}(\text{Dense}(F_{\text{global}}))$; // Final class probabilities

// Step 5: Explainability via GradCAM

$\mathsf{H} \leftarrow \text{GradCAM}(\hat{y}, F_{\text{cnn}})$; // Visualize regions influencing prediction

return $\hat{y}, \mathsf{H}$
