# Supplementary material for: Explainable transformer framework for fast cotton leaf diagnostics and fabric defect detection
Source: iScience. 2025 Dec 11;29(2):114411. doi: 10.1016/j.isci.2025.114411 (PMC12915276; doi:10.1016/j.isci.2025.114411)
Supplement: mmc 2: All suppliment Materials.pdf [file mmc2.pdf]

Algorithm S1. XCottL-FebViT for cotton leaf and fabric defect classification

Input: image $I \in R^{H \times W \times C}$

Output: predicted class label $\hat{y}$ and GradCAM explanation heatmap $\mathcal{H}$

Step 1: Preprocessing

1. $I_{\text{norm}} \leftarrow \text{Normalize}(I)$ // Min–max normalization
2. $I_{\text{aug}} \leftarrow \text{ApplyAugmentation}(I_{\text{norm}})$ // Dataset-specific augmentations

Step 2: Hierarchical CNN-based feature extraction

3. $F_0 \leftarrow \text{Conv3x3}(I_{\text{aug}})$ // Initial low-level features
4. For $(l = 1)$ to L_{cnn} do
   - $F_l \leftarrow \text{ConvBlock}(F_{l-1})$ // Stacked CNN layers to extract spatial features

   End for
5. $F_{\text{cnn}} \leftarrow F_{L_{\text{cnn}}}$

Step 3: LEViT transformer encoding

6. $T_0 \leftarrow \text{Tokenize}(F_{\text{cnn}})$ // Patch-wise token embedding
7. For $(l = 1)$ to L_{levit} do
   - a. $T_l \leftarrow \text{MHSA}(T_{l-1})$ // Multi-Head Self-Attention
   - b. $T_l \leftarrow \text{MLP}(T_l)$ // Feed-forward projection
   - c. If $\text{ShrinkLayer}(l)$ then
     - $T_l \leftarrow \text{Shrink}(T_l)$ // Spatial downsampling to reduce complexity

     End if

   End for
8. $F_{\text{levit}} \leftarrow T_{L_{\text{levit}}}$

Step 4: Classification

9. $F_{\text{global}} \leftarrow \text{GlobalAvgPool}(F_{\text{levit}})$ // Reduce to vector representation
10. $\hat{y} \leftarrow \text{Softmax}\left(\text{Dense}(F_{\text{global}})\right)$ // Final class probabilities

Step 5: Explainability via GradCAM

11. $\mathcal{H} \leftarrow \text{GradCAM}(\hat{y}, F_{\text{cnn}})$ // Visualize regions influencing prediction

Return $\hat{y}, \mathcal{H}$.

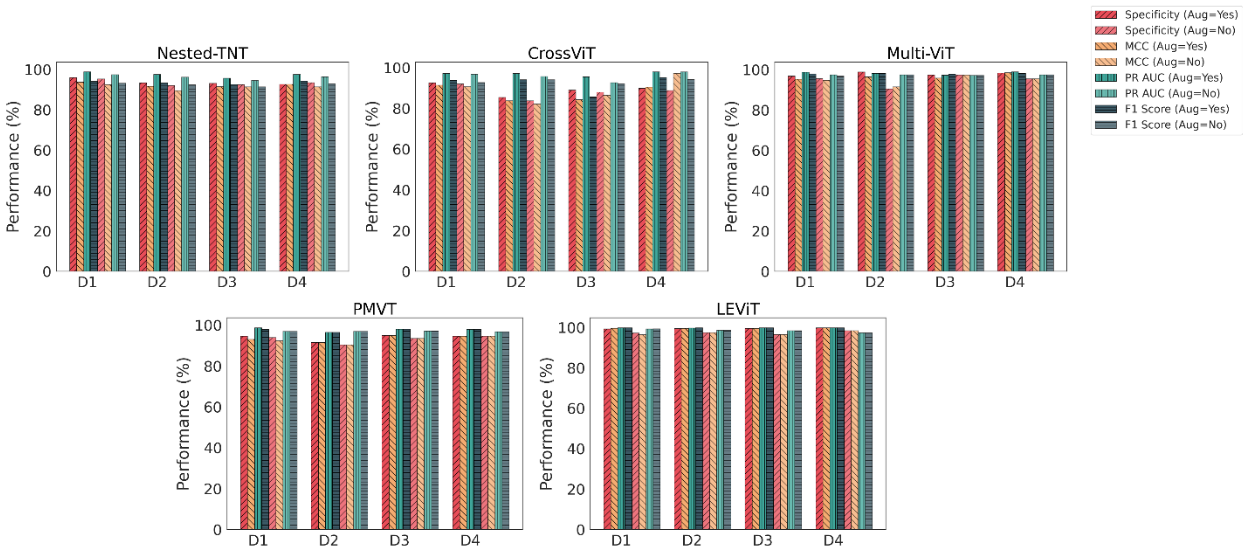

Figure S1. The bar chart compares five DL models on cotton and fabric defect datasets using Specificity, MCC, PR AUC, and F1 Score. Results show data augmentation improves performance, with LEViT achieving the best overall results.

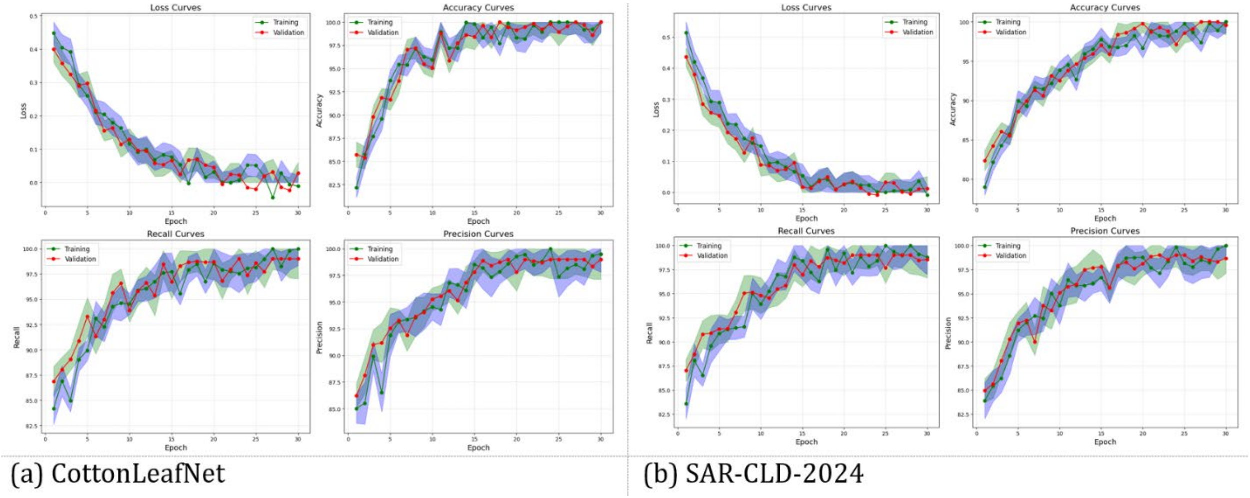

Figure S2. Learning curve of the LEViT model on balanced leaf datasets.

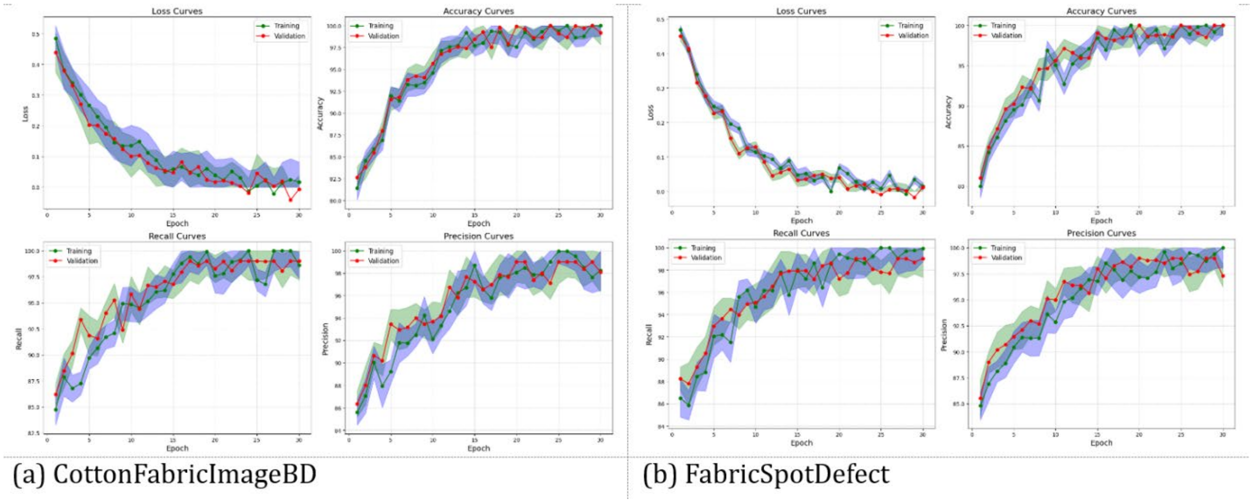

Figure S3. Learning curve of the LEViT model on balanced fabric datasets.

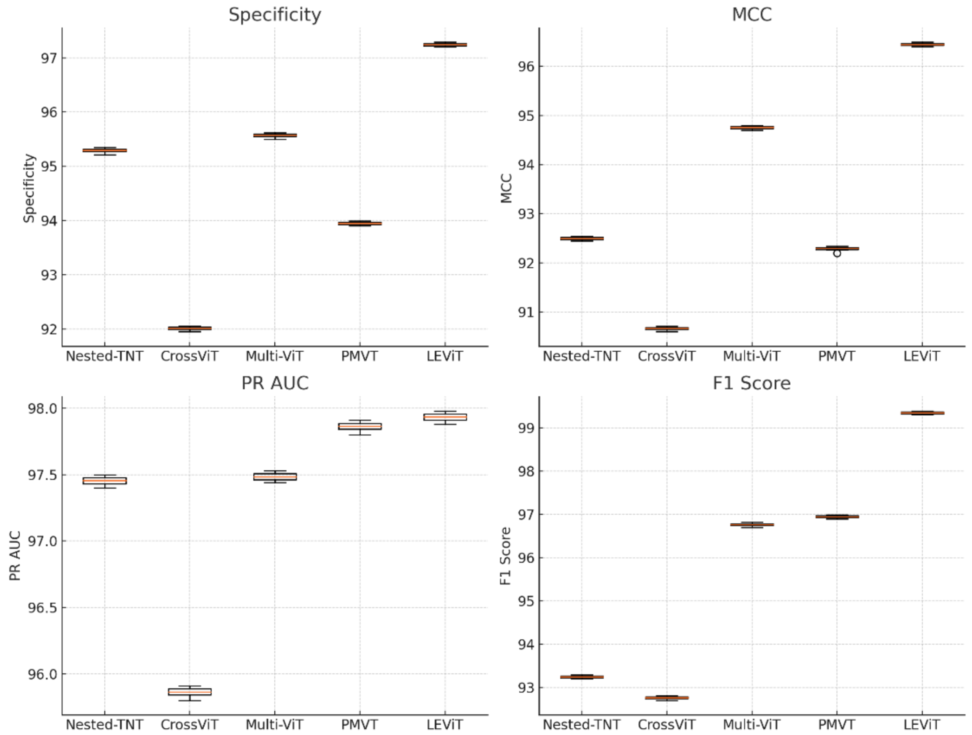

Figure S4. The evaluation figures depict different model types performing in 10-fold cross-validation using all evaluation metrics shown in boxplots. Studies show LEViT achieves the most consistent high median performance scores while showing minimal variability between measurements.

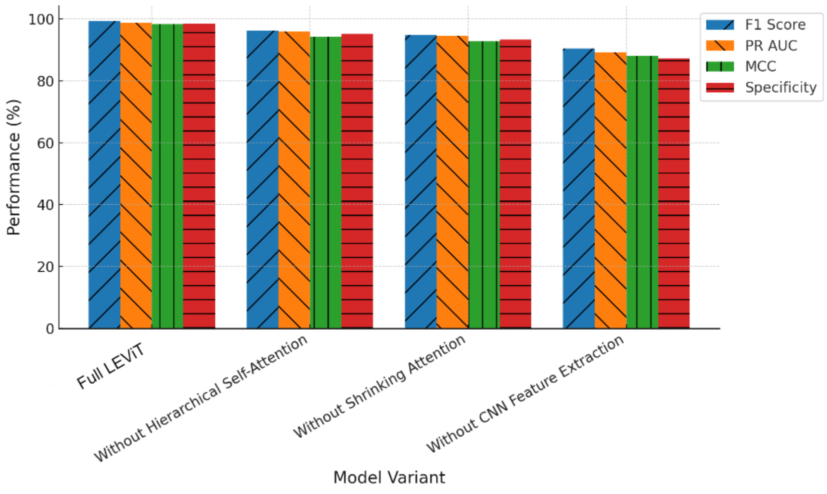

Figure S5. The bar chart demonstrates how LEViT's important elements affect performance where CNN Feature Extraction stands as the primary element followed by Shrinking Attention and Hierarchical Self-Attention elements.
